# Type VI secretion system completeness shapes evolutionary trade-offs in the *Acinetobacter baumannii* resistome

**DOI:** 10.3389/fmicb.2026.1867466

**Published:** 2026-07-15

**Authors:** Meng Zhang, Shuang Wang, Jinying Gao, Jing Jie, Qianxue Yu, Dan Li, Lei Song, Xiaoye Fan

**Affiliations:** State Key Laboratory for Diagnosis and Treatment of Severe Zoonotic Infectious Diseases, Department of Respiratory Medicine, Center of Pathogen Biology and Infectious Diseases, The First Hospital of Jilin University, Changchun, China

**Keywords:** *Acinetobacter baumannii*, antimicrobial resistance, conjugative plasmids, horizontal gene transfer, mobile genetic elements, natural transformation, type VI secretion system

## Abstract

The rapid global dissemination of multidrug-resistant *Acinetobacter baumannii* poses a critical threat to public health, yet the role of the Type VI Secretion System (T6SS)—a contact-dependent interbacterial weapon—in shaping the antimicrobial resistome remains poorly understood. Here, we integrated clinical metagenomics and large-scale comparative genomics to investigate the association between T6SS completeness and resistome organization. T6SS status was not independently associated with overall antimicrobial resistance genes (ARGs) burden or alpha diversity after controlling for shared evolutionary history and genomic background. However, T6SS completeness was associated with distinct resistome composition across multiple lineages. T6SS-complete genomes were preferentially enriched in chromosomally associated resistance determinants, including intrinsic *β*-lactamases and multidrug efflux systems, alongside tighter genomic co-localization between ARGs and mobile genetic elements (MGEs), consistent with localized chromosomal integration of resistance-associated mobile elements. This foundational prerequisite was supported by experimental validation of efficient T6SS-dependent interbacterial killing in a hyper-resistant lineage. Conversely, T6SS-incomplete genomes were significantly enriched in highly potent exogenously acquired ARGs, including *blaNDM-1* and *blaCTX-M*, frequently alongside structurally uncoupled MGEs. Together, these findings are consistent with an evolutionary trade-off model in which T6SS-complete and T6SS-incomplete *A. baumannii* populations exhibit distinct resistance acquisition strategies and contrasting genomic contexts of horizontal gene transfer, thereby contributing to divergent resistome organization.

## Introduction

The rapid global dissemination of multidrug-resistant (MDR) and extensively drug-resistant (XDR) *Acinetobacter baumannii* represents one of the most intractable challenges in modern healthcare. Thriving in high-stress clinical microenvironments, such as Intensive Care Units (ICUs), this opportunistic pathogen exhibits extraordinary genomic plasticity, rapidly acquiring antimicrobial resistance genes (ARGs) to survive potent antibiotic regimens (Smith et al., 2023; Vesel et al., 2023). Traditionally, the dissemination of ARGs has been largely attributed to independent horizontal gene transfer (HGT) events mediated by mobile genetic elements (MGEs), including plasmids (Weber et al., 2015; Di Venanzio et al., 2019), transposons (Tns)(Boral et al., 2025), and insertion sequences (ISs)(Zhang et al., 2026). However, the overarching ecological regulators and intrinsic structural constraints that shape how and why specific pathogenic lineages preferentially adopt distinct resistome architectures remain a critical blind spot in evolutionary microbiology.

Emerging evidence suggests that bacterial protein secretion systems—specifically the Type VI Secretion System (T6SS)—may serve as central regulatory hubs for these evolutionary dynamics (Jie et al., 2026). Initially characterized as a phage-derived contractile secretion system utilized for contact-dependent microbial antagonism, the T6SS has recently been increasingly implicated in the indirect regulation of antimicrobial resistance (Ma and Mekalanos, 2010; Basler et al., 2013). Beyond mere interbacterial warfare, T6SS-mediated predation lyses neighboring competitor cells, releasing extracellular DNA (eDNA) that can subsequently be incorporated into the predator’s genome via natural transformation, thereby actively facilitating HGT through an endogenous pathway (Winter et al., 2021).

Paradoxically, this predatory behavior fundamentally conflicts with other avenues of genetic acquisition, particularly via conjugative transfer. The uptake of large conjugative plasmids carrying formidable ARGs (such as carbapenemases) frequently coincides with the active repression or disruption of the host’s chromosomal T6SS. Previous studies have proposed that plasmid-mediated silencing may represent an adaptive strategy to prevent the lethal targeting of donor cells during the intimate cell-to-cell contact required for conjugation(Weber et al., 2016; Di Venanzio et al., 2019; Yadav et al., 2021; Godeux et al., 2022; He et al., 2025). These observations suggest a potential evolutionary trade-off at the intersection of interbacterial competition and exogenous genetic acquisition (Weber et al., 2015; Di Venanzio et al., 2019; York, 2019). While experimental approaches are essential for elucidating T6SS expression and activity, such analyses are inherently limited in scalability and are highly dependent on specific environmental and physiological conditions. Consequently, genome-resolved analyses offer a powerful approach to investigate the relationship between T6SS architecture and resistome organization. Yet, large-scale, population-level genomic evidence linking T6SS genetic organization to resistome architecture remains limited.

In this study, we hypothesize that the genetic completeness of the T6SS is not merely a structural virulence-associated feature, but may also be evolutionarily associated with distinct evolutionary trade-offs in resistome composition and mobilization patterns of *A. baumannii*. To test this, we first mapped the real-world co-occurrence networks of the *Acinetobacter* resistome using extensive clinical metagenomic next-generation sequencing (mNGS) profiles from 873 samples. Subsequently, we conducted a systematic comparative genomic analysis of 2,915 *A. baumannii* genomes. By integrating mobilome spatial profiling, plasmid network analysis, and phylogenomic mapping, we uncovered two starkly divergent evolutionary trajectories. Finally, we validated the foundation of our predictive model by confirming the potent *in vitro* antibacterial activity of T6SS derived from a hyper-resistant global lineage. Our integrated computational and experimental approach provides a comprehensive framework for understanding how interbacterial weaponry shapes the evolutionary trajectory of antibiotic-resistant pathogens.

## Results

### Clinical mNGS profiles reveal highly interconnected *Acinetobacter* resistome networks in high-stress microenvironments

**Figure 1:**
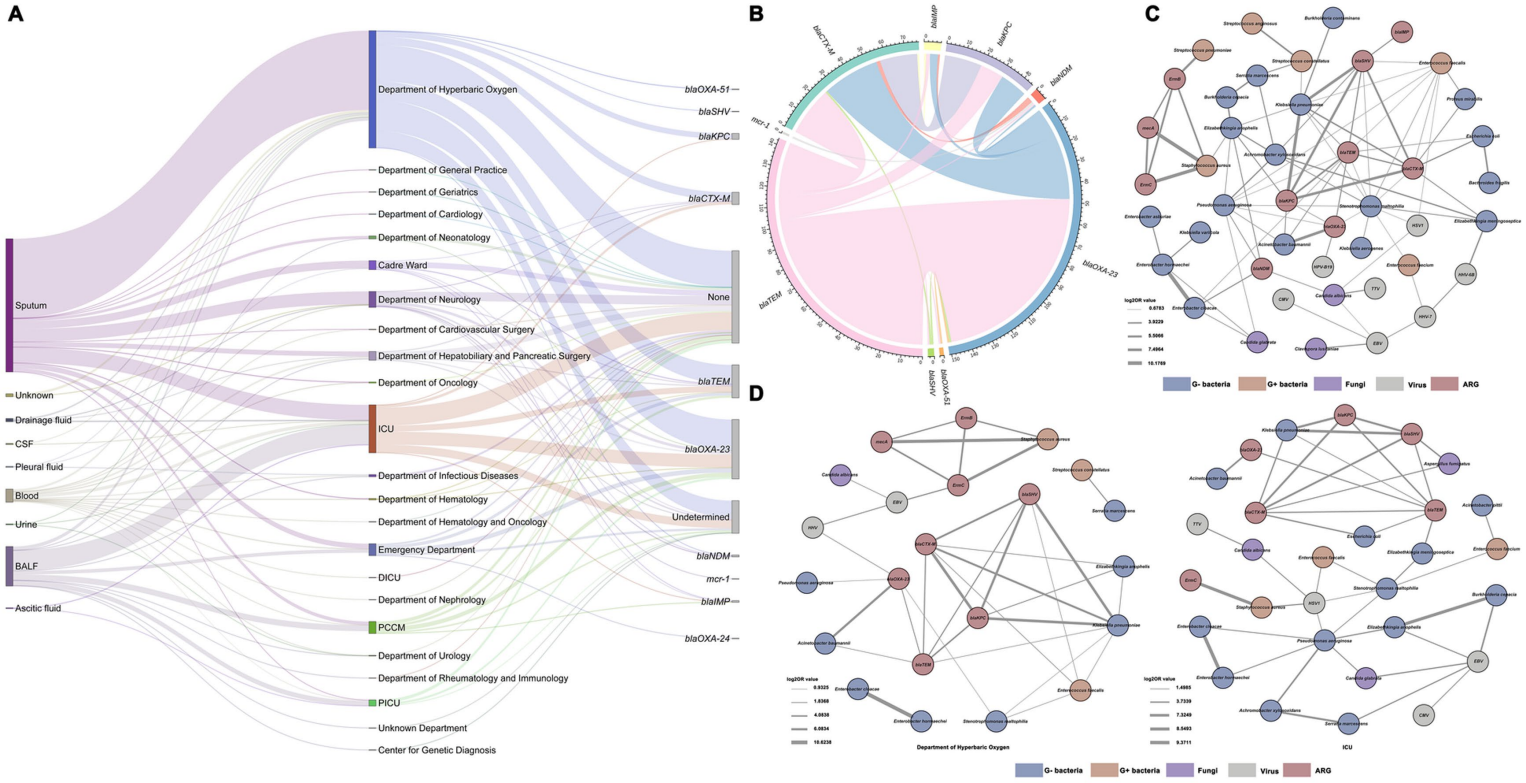
Clinical distribution and co-occurrence patterns of *Acinetobacter*-associated ARGs. **(A)** Sankey diagram illustrating relationships among clinical sample sources (left), hospital departments (middle), and detected ARGs (right) based on mNGS data. Node size is proportional to the number of its connected neighbors, while the width of the flows (ribbons) represents the relative quantity of isolates. **(B)** Chord diagram depicting pairwise co-occurrence relationships among ARGs detected in *A. baumannii*. Ribbon width is proportional to co-occurrence frequency. **(C)** Hospital-wide microbial–ARG co-occurrence network constructed from 873 clinical samples. Edges represent statistically significant co-occurrence relationships, with edge thickness proportional to the log2(odds ratio). **(D)** Department-specific microbial-ARG co-occurrence subnetworks for the Department of Hyperbaric Oxygen Therapy and the ICU, illustrating department-level heterogeneity in co-occurrence patterns.

To elucidate the real-world epidemiological distribution and ecological co-occurrence of *Acinetobacter* and its ARGs, we retrospectively analyzed 873 *Acinetobacter*-positive mNGS samples, of which 738 were positive for *A. baumannii* (Supplementary Table S1). A Sankey diagram mapped the flow of specimens across diverse clinical departments, highlighting the ubiquitous presence of *blaOXA-23* and *blaTEM* (Figure 1A). Subsequent co-occurrence analysis confirmed that *blaOXA-23* and *blaTEM* were the most prevalent resistance determinants, frequently co-existing within identical clinical samples (Figure 1B).

To quantitatively dissect these microbial interactions, we constructed a co-occurrence network utilizing Fisher’s exact test with false discovery rate correction (adjusted *p* < 0.05, *OR* > 1). Significant positive associations were identified between *A. baumannii* and key resistance genes. Notably, *blaOXA-23* showed an extreme enrichment pattern (*OR* = 180.56, 95%CI: 11.19–2913.05, *p*_adj_ < 0.001), indicating a strong association likely driven by its distribution within dominant *A. baumannii* lineages. In contrast, *blaKPC* (*OR* = 4.34, 95%CI: 1.20–15.61, *p*_adj_ = 0.040) and *blaTEM* (*OR* = 3.66, 95%CI: 2.17–6.19, *p*_adj_ < 0.001) were moderately associated with *A. baumannii*. Additionally, *A. baumannii* showed a significant co-occurrence pattern with *Pseudomonas aeruginosa* (*OR* = 2.76, 95%CI: 1.43–5.31, *p*_adj_ = 0.0067), suggesting intense interspecific interactions in shared niches (Figure 1C). Department-wise distribution analysis further revealed that the Intensive Care Unit (ICU) and the Department of Hyperbaric Oxygen harbored the highly connected and complex ARG networks. In these high-stress environments, characterized by severe antibiotic selective pressure and robust oxidative stress, *blaKPC*, *blaSHV*, *blaTEM*, and *blaCTX-M* formed tightly linked hubs (Figure 1D). The exceptional topological complexity of the ICU network reflects frequent polymicrobial infections and may indicate conditions favorable to HGT at the population level.

### T6SS status was not associated with a significant difference in total ARG burden

Given the complex ARG networks observed clinically, we hypothesized that the T6SS might play a crucial role in shaping the resistome. After stringent quality control, 2,946 high-quality genomes were retained. Among these, 31 genomes with ambiguous T6SS profiles due to potential assembly artifacts at contig boundaries were excluded. A total of 2,915 genomes were therefore used for downstream analyses and were classified into a T6SS-complete group (*n* = 2,480) and a T6SS-incomplete group (*n* = 435).

**Figure 2:**
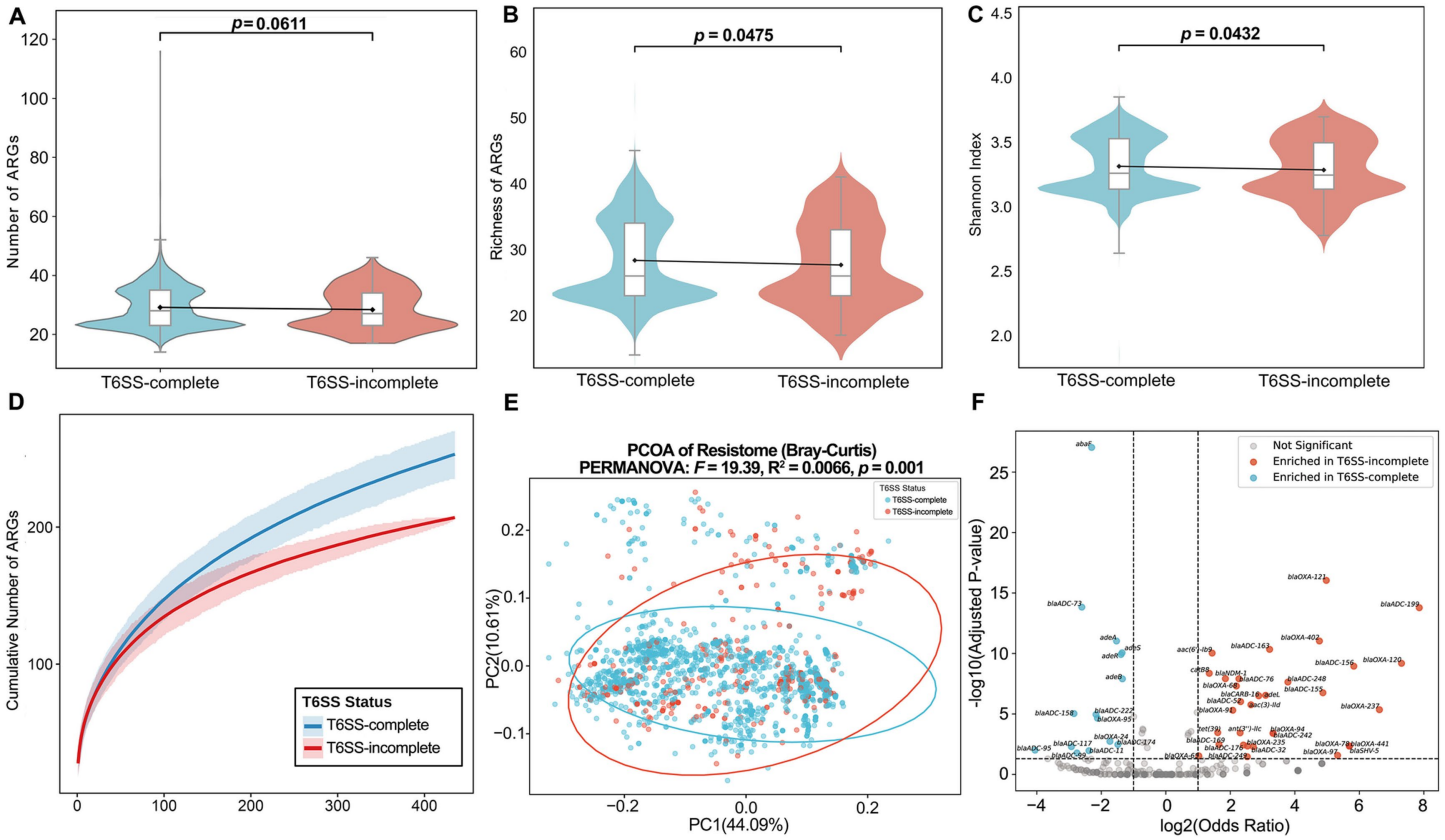
Comparative analysis of ARG profiles between T6SS-complete and T6SS-incomplete *A. baumannii* genomes. **(A–C)** Genomic ARG profiles characterized by **(A)** total abundance, **(B)** ARG richness (unique ARG types), and **(C)** Shannon diversity. Violin plots illustrate data distribution, with internal boxplots representing the median and interquartile range (IQR). Statistical significance was determined by the Wilcoxon rank-sum test. **(D)** Rarefaction analysis of cumulative ARG richness. The T6SS-complete group was randomly subsampled to match the size of the T6SS-incomplete group (*n* = 435), with 95% confidence intervals estimated from 1,000 bootstrap iterations. **(E)** PCoA based on Bray–Curtis’ dissimilarity show the compositional differences of ARGs between the two groups. Ellipses indicate the 95% confidence interval. Group separation was assessed by PERMANOVA. **(F)** Differential enrichment of ARGs between T6SS states based on Fisher’s exact tests. ARGs significantly enriched in T6SS-incomplete genomes are shown in red, while those enriched in T6SS-complete genomes are shown in blue. Dashed lines indicate significance and effect-size thresholds.

Total ARG burden per genome was comparable between groups (*p* = 0.0611), with a negligible effect size (Cliff’s delta = 0.056) and a median difference of only 1 ARG (95% bootstrap CI: −1 to 3) (Figure 2A). Similarly, neither the abundance nor diversity of carbapenem resistance determinants per genome differed significantly between groups (*p* > 0.05) (Supplementary Figures 1A,B). To account for potential confounding factors, including genome size, assembly quality, and phylogenetic non-independence, we further performed a phylogenetically informed PGLS analysis. Consistent with the univariate results, T6SS status was not significantly associated with total ARG burden (*β* = −0.29, *p* = 0.591; *λ* = 0.898), whereas genome size remained the strongest predictor of ARG burden (*p* < 2 × 10^−16^). Collectively, these findings indicate that overall ARG burden is largely explained by genome background rather than T6SS status.

### Intact T6SS is associated with distinct resistome organization and differential enrichment of intrinsic ARGs

#### ARG alpha diversity is not independently associated with T6SS status

Although total ARG burden was comparable between groups, we next examined whether T6SS status was associated with genome-level ARG diversity. Initial univariate analyses suggested modest differences in both ARG richness (*p* = 0.0475; Figure 2B) and Shannon diversity (*p* = 0.0432; Figure 2C) between the groups. However, effect size estimates were negligible (Cliff’s ≈ 0.06 for both comparisons), indicating limited biological magnitude. Importantly, PGLS models revealed that neither ARG richness (*p* = 0.943; Pagel’s *λ* = 0.962) nor Shannon diversity (*p* = 0.896; Pagel’s λ = 0.953) remained independently associated with T6SS status after phylogenetic correction. These results suggest that the modest differences in ARG richness and alpha diversity are largely attributable to phylogenetic background and genome-scale covariates rather than T6SS status itself.

#### Population-level ARG accumulation

Rarefaction curve analysis, normalized to an equal sampling depth of 435 genomes with 1,000 bootstrap iterations, further highlighted distinct patterns of ARG accumulation. The T6SS-complete consistently exhibited greater cumulative ARG richness than the T6SS-incomplete, indicating a broader and more heterogeneous ARG repertoire. Notably, the rarefaction curve of the T6SS-complete group did not reach saturation within the current sampling range, suggesting the continued presence of low-frequency ARGs. In contrast, the T6SS-incomplete group approached a plateau more rapidly, consistent with a comparatively restricted ARG repertoire (Figure 2D).

#### Resistome composition differs between T6SS states

Principal Coordinates Analysis (PCoA) based on Bray-Curtis’ dissimilarity revealed a statistically significant but modest separation between groups (PC1: 44.09%, PC2: 10.61%; PERMANOVA: *F* = 19.39, R^2^ = 0.0066, *p* = 0.0010) (Figure 2E). Although the effect size was small, this is consistent with high-dimensional resistome variation. Notably, PERMDISP analysis revealed significant differences in multivariate dispersion between groups (*F* = 16.53, *p* < = 0.001), with the T6SS-incomplete population exhibiting greater dispersion in resistome composition, indicating increased within-group variation (Supplementary Figure 1C). To control for potential confounding by clonal population structure, we further performed a lineage-stratified PERMANOVA restricted to STs containing both T6SS states. The association between T6SS status and resistome composition remained significant after lineage control (stratified PERMANOVA: *F* = 16.32, R^2^ = 0.0080, *p* = 0.001), indicating that the observed resistome differentiation was not solely attributable to lineage composition.

#### Differentially enriched resistance determinants

Feature-level analysis via Fisher’s exact test revealed distinct ARG enrichment patterns. The T6SS-complete group was preferentially enriched in chromosomally associated or intrinsic resistance determinants, including *blaADC* cephalosporinases, *blaOXA-24*/*95*, *abaF* and RND-type efflux pump (*adeA* and *adeB*). In contrast, the T6SS-incomplete group was enriched in highly potent, exogenously acquired ARGs, including *blaNDM-1*, *blaSHV-5*, and multiple aminoglycoside-modifying enzymes. (Figure 2F).

#### Sensitivity analyses controlling for lineage effects

To assess the potential influence of phylogenetic non-independence and clonal expansion, we performed a series of sensitivity stratification analyses. Firstly, several major differentially enriched ARGs, including *adeA*, *adeB*, *adeR*, *adeS*, *abaF*, *ant(3″)-IIc*, and *adeL*, were detected across multiple sequence types rather than being restricted to a single lineage (Supplementary Figure 2A). Secondly, after exclusion of all ST2 genomes (*n* = 939; 32.2% of total genomes), the rest comprised 1,976 genomes, including 1,667 T6SS-complete and 309 T6SS-incomplete genomes. The association between T6SS status and resistome composition remained significant (PERMANOVA: *F* = 23.57, R^2^ = 0.0118, *p* < = 0.001; PC1: 24.83%, PC2: 15.36%), and differential ARG enrichment patterns were broadly consistent with the primary analysis (Supplementary Figures 2B,C). Thirdly, lineage-aware stratified approach was conducted within two most dominant lineages, ST2 and ST499 (Supplementary Figures 3A,B). PERMANOVA confirmed significant differentiation in both ST2 (PC1: 33.78%, PC2: 28.84%; *F* = 38.53, R^2^ = 0.0395, *p* < = 0.001) and ST499 (PC1: 52.28%, PC2: 27.38%; *F* = 4.07, R^2^ = 0.0218, *p* = 0.014), and PERMDISP tests were both non-significant (*p* > 0.05). Collectively, these analyses indicate that the observed resistome differentiation cannot be explained solely by lineage composition or expansion of dominant clones.

### Distinct mobilome-resistome architectures reflect alternative routes of HGT

**Figure 3:**
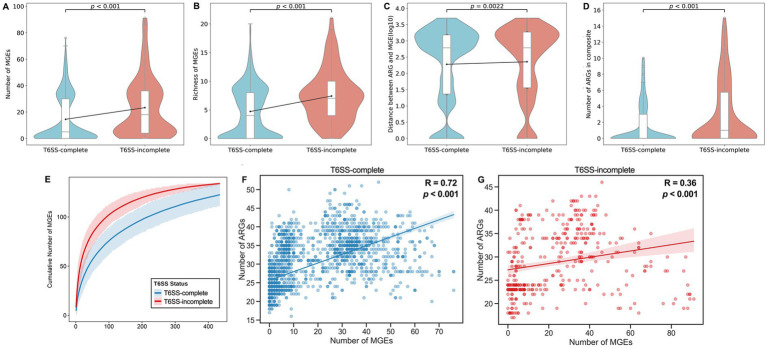
Genomic association between MGEs and ARGs. **(A–D)** Comparison of genome-level MGE and ARG-associated features between T6SS-complete (teal) and T6SS-incomplete (coral) *A. baumannii* genomes, including **(A)** total MGE count per genome, **(B)** MGE richness (unique MGE types), **(C)** log10-transformed genomic distance between each ARG and its nearest MGE, and **(D)** number of ARGs located within composite transposons. Violin plots depict the data distribution, with internal boxplots indicating the median and IQR. **(E)** Rarefaction curves of MGE richness. The T6SS-complete group was randomly subsampled to match the size of the T6SS-incomplete group (*n* = 435), with 95% confidence intervals derived from 1,000 bootstrap iterations. (F, G) Correlations between genome-level ARG and MGE counts in the **(F)** T6SS-complete and **(G)** T6SS-incomplete groups. Spearman’s rank correlation coefficients were calculated to assess associations between variables. Shaded areas represent 95% confidence intervals.

To further investigate these compositional differences, we compared mobilome architectures between two groups. Paradoxically, T6SS-incomplete strains harbored significantly more total and diverse MGEs than T6SS-complete strains (*p* < 0.001) (Figures 3A,B). However, evaluating the physical proximity between ARGs and their nearest MGEs (< 5 kb threshold) revealed distinct probability density distributions. The complete group possessed a higher density of ARG-MGE pairs at shorter genomic distances, implying a tighter spatial coupling consistent with more localized ARG-MGE associations (Figure 3C). We further examined ARGs embedded within composite transposons. The T6SS-incomplete group harbored significantly more ARGs within composite transposons, indicating a greater contribution of transposon-associated resistance cargo to the resistome architecture of these genomes (Figure 3D, *p* < 0.001). The collective MGEs diversity was further evaluated through rarefaction analysis. At given sampling depth (*n* = 435 genomes), the cumulative number of unique MGE types in the T6SS-incomplete group expanded at a substantially steeper rate compared to the T6SS-complete group (Figure 3E). The rarefaction curves indicate that T6SS-incomplete genome harbor a broader repertoire of mobile genetic elements at the population level.

This architectural difference was further supported by Spearman correlation analysis. In the T6SS-complete group, MGEs abundance exhibited a significant positive correlation with ARG counts (*R* = 0.72, *p* < 0.001). This association underscores a tight coupling between resistance determinants and mobile genetic elements at the genome level (Figure 3F). Conversely, the T6SS-incomplete group showed a substantially weakened correlation (*R* = 0.36, *p* < 0.001), suggesting that variation in MGE abundance is less tightly coupled to ARG content in these genomes (Figure 3G).

Together, these results support the existence of distinct mobilome–resistome architectures associated with T6SS status.

### Distinct plasmid mobility profiles characterize T6SS-incomplete genomes

**Figure 4:**
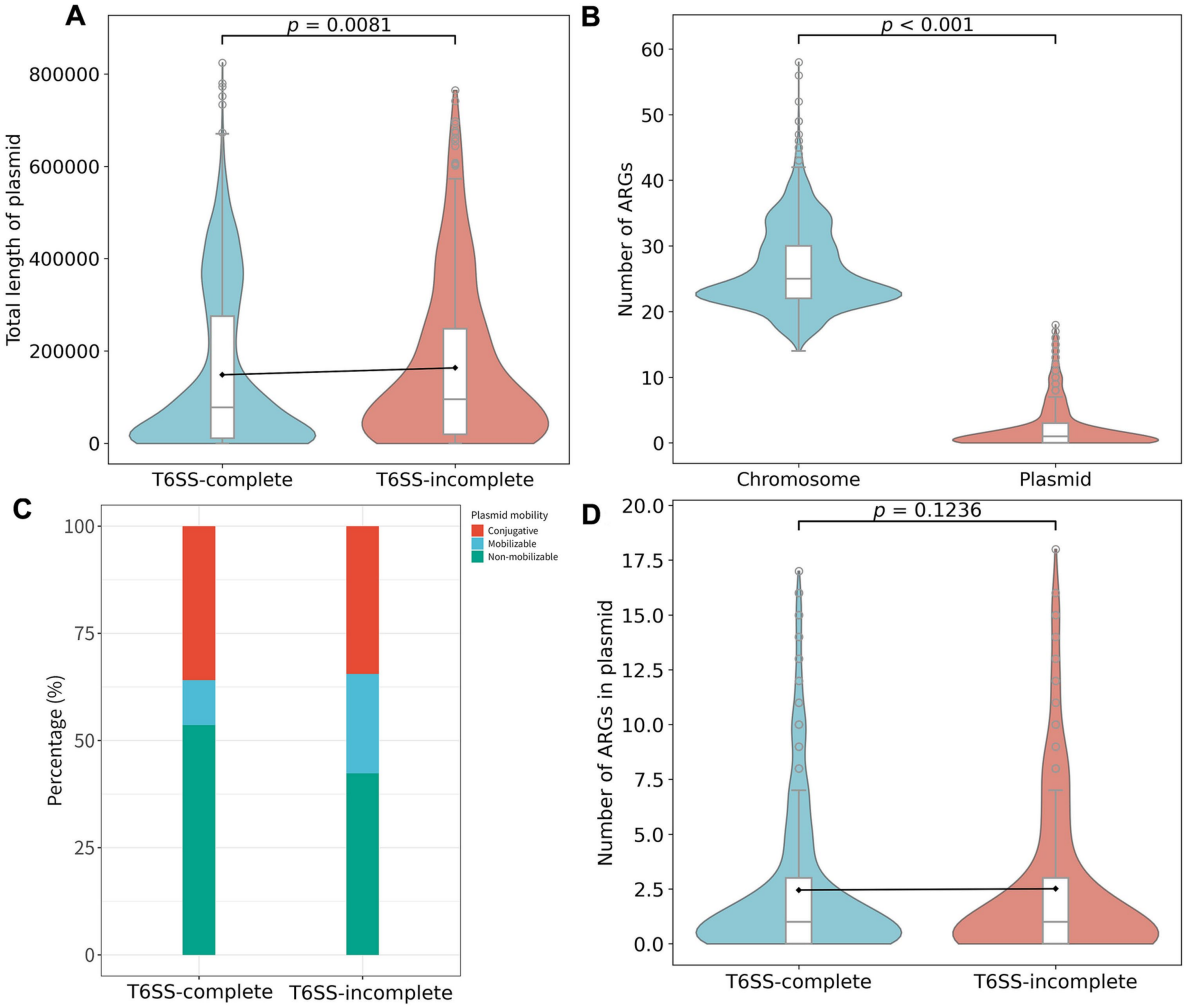
Comparative analysis of plasmid content, mobility, and plasmid-associated ARGs across T6SS states. **(A)** Total predicted plasmid length per genome in T6SS-complete and T6SS-incomplete genomes. Violin plots showing the distribution, with embedded boxplots indicating the median and IQR. **(B)** Genomic localization of ARGs, showing the distribution of chromosomal and plasmid-borne resistance determinants. **(C)** Proportional distribution of plasmid mobility types in plasmid-positive genomes. Plasmids were classified as conjugative, mobilizable, or non-mobilizable for each group. **(D)** Number of plasmid-borne ARGs per plasmid-positive genome in the two T6SS groups.

The T6SS-incomplete strains contained a significantly larger total length of predicted plasmids (Figure 4A). Predicted plasmid sequences were detected in 2,100 of 2,480 T6SS-complete genomes (84.68%) and 380 of 435 T6SS-incomplete genomes (87.36%), indicating that plasmid carriage was common in both groups. Across all genomes, ARGs were predominantly encoded on chromosomes rather than plasmids (*p* < 0.01) (Figure 4B), indicating that the majority of ARGs were stably integrated into bacterial chromosomes. Among plasmid-positive genomes, the number of plasmid-borne ARGs did not differ significantly between T6SS-complete and T6SS-incomplete strains (median = 1 in both groups, *p* = 0.1236; Figure 4D). In contrast, marked differences were observed in plasmid mobility profiles (Figure 4C). T6SS-complete genomes were predominantly associated with non-mobilizable plasmids (1,126/2,100, 53.62%), whereas mobilizable plasmids were less frequent 10.43% (219/2,100). T6SS-incomplete genomes exhibited a proportion of mobilizable plasmids (88/380, 23.16%) and a corresponding reduction in non-mobilizable plasmids (161/380, 42.37%), while conjugative plasmids remained comparable between groups (131/380, 34.47% vs. 35.95%,755/2,100). These results indicate a shift in plasmid mobility profiles between the two T6SS states.

Importantly, conjugative plasmids likely function as large genetic vehicles carrying exogenous ARGs (as identified in Figure 2F) together with extensive accessory genes, which may contribute to the expansion of the mobilome observed in T6SS-incomplete strains.

### Phylogenomic mapping links T6SS status to lineage-associated resistome patterns

**Figure 5:**
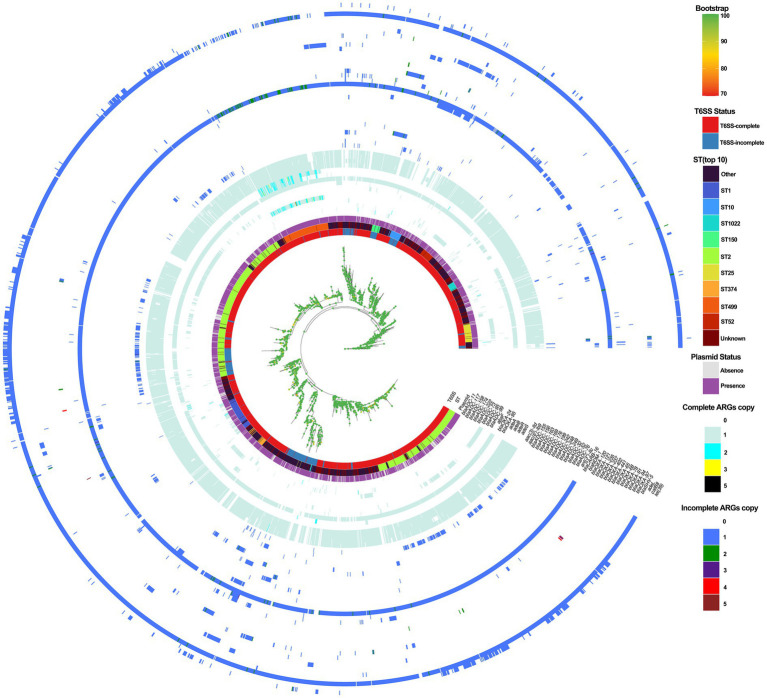
Phylogenetic distribution of T6SS status and associated genomic features in *A. baumannii*. The maximum-likelihood phylogenetic tree illustrates the evolutionary relationships among the study population. Annotations from the innermost to the outermost ring represent: T6SS status (Complete, red; Incomplete, blue), sequence type (ST, top10), plasmid presence/absence, differentially enriched ARG. The bootstrap values (70–100%) are indicated at the internal nodes.

To synthesize our multi-omics findings into an evolutionary framework, we constructed a core-genome phylogenetic tree annotated with T6SS status, sequence types (STs), plasmid carriage, and differentially enriched ARGs (Figure 5). The distribution of T6SS status is not entirely random across the phylogeny. While T6SS-incomplete strains are widely dispersed across the phylogeny, they also exhibit distinct clustering within specific evolutionary lineages. This pattern suggests that the loss or disruption of T6SS may be associated with clade-specific evolutionary events. Importantly, this clustering does not strictly correspond to STs, as strains within the same ST can still display different T6SS states. Plasmid-bearing strains (*n* = 2,480; 85.08%) are widely distributed throughout the phylogeny, and no clear phylogenetic clustering is observed. No clear visual association between plasmid distribution and T6SS status can be reliably inferred from the phylogenetic context alone.

### T6SS-mediated interbacterial killing and effector toxicity in a hyper-resistant *A. baumannii* lineage

**Figure 6:**
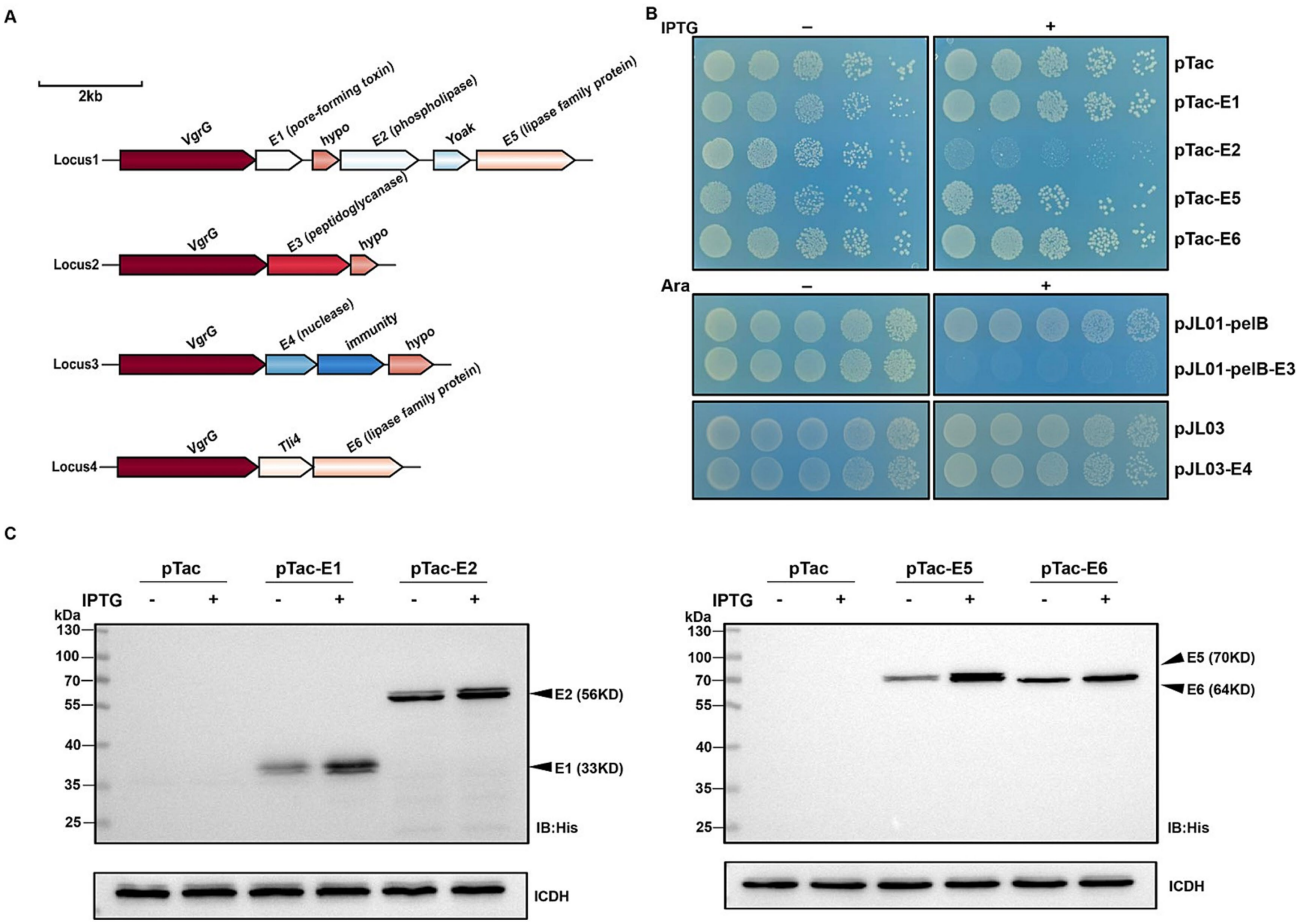
Genomic organization and *in vitro* toxicity validation of candidate T6SS effectors in multidrug-resistant *A. baumannii* strain AB308. **(A)** Genetic organization of the *vgrG* related locus in AB308 and annotation of six candidate effector proteins (E1–E6). **(B)** Spotting viability assays to assess the toxicity of candidate effectors (E1–E6) in *E. coli* DH5α. Cultures were normalized to an initial OD_600_nm of 1.0, followed by serial 5-fold dilutions and spotting onto LB agar plates. Growth was compared under non-inducing (−) and inducing conditions (+). Empty vectors (pTac, pJL03, and pJL01-pelB) served as negative controls. **(C)** Verification of heterologous protein expression of candidate effectors in *E. coli*. Western blot was performed using anti-His-tag antibodies to detect the expression of E1, E2, E5, and E6 in *E. coli* DH5α. Lanes marked with “−” and “+” represent samples from uninduced group and induced group, respectively. The arrows indicate the predicted band size.

The foundational prerequisite of our “kill-and-scavenge” hypothesis is that the T6SS in these hyper-resistant lineages functions as a genuine antibacterial weapon capable of liberating eDNA. To experimentally validate this, we selected strain AB308, a multidrug-resistant clinical isolate recovered from a sputum specimen at our hospital in 2023. AB308 was selected because it harbors a structurally complete T6SS alongside a high burden of ARGs (Table 1). Six putative effector proteins were identified: a DUF4123-containing pore-forming toxin (E1), the phospholipase effector Tle1 (E2), a peptidoglycanase (E3), a nuclease (E4), and two lipase family proteins (E5 and E6) (Figure 6A).

**Table 1:** The distribution of ARGs in the clinical strain AB308 genome.

ARG	Resistance mechanism	Antibiotic classes
*blaADC-154*	Antibiotic inactivation	Cephalosporin
*blaOXA-132*	Antibiotic inactivation	Penicillin Beta-Lactam, carbapenem
*ant(3″)-IIc*	Antibiotic inactivation	Aminoglycoside
*lpsB*	Reduced permeability to antibiotic	Peptide antibiotic
*parC*	Antibiotic target alteration	Fluoroquinolone
*rsmA*	Antibiotic efflux	Phenicol, diaminopyrimidine, fluoroquinolone
*adeH*	Antibiotic efflux	Tetracycline, fluoroquinolone
*adeF*	Antibiotic efflux	Tetracycline, fluoroquinolone
*adeG*	Antibiotic efflux	Tetracycline, fluoroquinolone
*adeL*	Antibiotic efflux	Tetracycline, fluoroquinolone
*adeS*	Antibiotic efflux	Tetracycline, glycylcycline
*adeR*	Antibiotic efflux	Tetracycline, glycylcycline
*adeA*	Antibiotic efflux	Tetracycline, glycylcycline
*adeB*	Antibiotic efflux	Tetracycline, glycylcycline
*abaQ*	Antibiotic efflux	Fluoroquinolone
*abaF*	Antibiotic efflux	Phosphonic Acid
*abeS*	Antibiotic efflux	Aminocoumarin, macrolide
*amvA*	Antibiotic efflux	Disinfecting agents and antiseptics, macrolide
*abeM*	Antibiotic efflux	Disinfecting agents and antiseptics, fluoroquinolone
*adeI*	Antibiotic efflux	MDR (Broad-spectrum)
*adeJ*	Antibiotic efflux	MDR (Broad-spectrum)
*adeK*	Antibiotic efflux	MDR (Broad-spectrum)
*adeN*	Antibiotic efflux	MDR (Broad-spectrum)

To determine whether the T6SS of AB308 is functionally active, we constructed a T6SS-deficient mutant by deleting the key structural gene *tssM*. Hcp secretion was readily detected in wild-type AB308 but was completely absent in the Δ*tssM* mutant, confirming active T6SS assembly and secretion in the wild-type strain (Supplementary Figure 4C). Consistently, wild-type AB308 exhibited strong antibacterial activity against *E. coli* in spot competition assays, quantitative CFU measurements, and prey growth-curve, whereas the Δt*ssM* mutant largely lost this killing capacity (Supplementary Figures 4A,B). These data firmly demonstrate that AB308 possesses a highly active T6SS capable of mediating efficient interbacterial killing.

We next evaluated the toxicity of the six candidate effectors (E1–E6) by heterologous expression in *E. coli* DH5α. Quantitative growth curves demonstrated that E2 and E3 caused severe growth inhibition. This was perfectly mirrored by the significant lack of colony clearance in spot viability assays. E5 exhibited moderate toxicity, whereas E1, E4, and E6 showed no overt toxicity under the tested conditions, with growth curves tracking closely with control (Supplementary Figure 5; Figure 6B). Western blot confirmed inducible expression of E1, E2, E5, and E6, with low basal expression observed in some uninduced controls, likely due to promoter leakiness (Figure 6C). Although E3 and E4 were not detected by Western blot, Coomassie Brilliant Blue staining confirmed successful sample loading (Supplementary Figure 6). The pronounced toxicity of E3 strongly suggests that biologically active protein was produced despite its failure to be detected. By contrast, E4 neither produced a detectable protein signal nor exhibited measurable toxicity under the tested conditions. Consequently, it remains unclear whether E4 lacks antibacterial activity or was not successfully expressed in the heterologous host. Its biological function therefore requires further investigation.

Collectively, these results demonstrate that AB308 harbors a structurally complete and functionally active T6SS capable of mediating efficient interbacterial killing. The identification of toxic effectors, particularly E2 and E3, further supports the antibacterial potential of this system and provides experimental support for the proposed “kill-and-scavenge” model in hyper-resistant lineages.

## Discussion

In the relentless arms race against clinical antimicrobial therapies, bacterial pathogens constantly optimize their genetic repertoires to survive under intense selective pressure (Elbaiomy et al., 2025). By integrating large-scale clinical mNGS profiling, comparative genomics, and *in vitro* functional validation, our study reveals that T6SS completeness is not associated with the overall burden of ARGs, but rather with distinct resistome architectures and mobilome configurations. We propose an evolutionary trade-off model comprising two mutually exclusive adaptive pathways: the T6SS-dependent “kill-and-scavenge” strategy and the T6SS-independent “plasmid-receptive” strategy.

For T6SS-complete strains, the observed mobilome-resistome organization is consistent with diversification patterns that may arise in environments where frequent interbacterial antagonism promotes DNA release and HGT. Although genome-level ARG burden and alpha diversity were not independently associated with T6SS status after phylogenetic correction, rarefaction analysis revealed a broader population-level ARG repertoire in T6SS-complete genomes. Moreover, these were preferentially enriched in chromosomally associated resistance determinants, including *blaADC* alleles, *blaOXA* variants, and *ade* efflux (Figure 2). Importantly, our *in vitro* experiments confirmed that representative T6SS-complete strains possess a functionally active T6SS that mediates efficient interbacterial killing (Supplementary Figure 4), and encode toxic effector candidates that further support their antibacterial potential (Figure 6), providing experimental support for the ecological plausibility of a kill-and-scavenge model. In polymicrobial niches, T6SS-mediated antagonism may release a continuous supply of eDNA from closely related strains, and subsequently uptake of this eDNA via natural transformation, and potentially, recombination-mediated genetic exchange (Ringel et al., 2017; Lin et al., 2019). Natural transformation typically involves the uptake of smaller DNA fragments, perfectly explaining why the MGEs in the complete group are tightly coupled in spatial proximity to ARGs and are highly predictive of the ARG counts (*R* = 0.72) (Figure 3). Thus, functional T6SS-complete strains act as predatory competitors, acquiring specific, small-scale genetic variants from eDNA released by lysed neighboring bacteria to optimize their intrinsic resistance without the metabolic burden of massive plasmids.

In contrast, T6SS-incomplete genomes displayed a markedly different genomic configuration. These strains harbored significantly larger plasmid complements, a broader mobilome repertoire, and a higher abundance of ARGs embedded within composite transposons (Figures 3, 4). At the gene level, they were enriched in clinically important acquired ARGs, including *blaNDM*, *blaSHV*, and multiple aminoglycoside-modifying enzymes (Figure 2). Although most ARGs across the dataset were chromosomally encoded (Figure 4B), this does not diminish the importance of plasmid-mediated HGT. Chromosomal ARGs likely represent the stable core resistome, whereas plasmids primarily act as mobile vehicles that introduce novel ARGs and associated accessory genetic elements into recipient populations (Azour et al., 2026). Consequently, plasmid dynamics may substantially influence resistome diversification even when the majority of ARGs ultimately reside on chromosomes. Previous molecular studies have demonstrated that large conjugative plasmids (LCPs) originating from other species frequently encode transcriptional repressors (such as TetR-family proteins) designed to actively silence the recipient’s chromosomal T6SS (Di Venanzio et al., 2019; Benomar et al., 2021; Flaugnatti et al., 2025; He et al., 2025). This ensures that the bacterial donor is not killed during conjugation. Because these LCPs also carry substantial amounts of accessory DNA and complex composite transposons, the total count of MGEs inherently inflates (Godeux et al., 2022). This genomic architecture is consistent with the massive influx of uncoupled MGEs and the weak correlation (*R* = 0.36) observed in the incomplete group (Figure 3G). Rather than representing alternative levels of resistance, the two T6SS states therefore appear to be associated with distinct routes of ARG acquisition and dissemination.

These divergent genomic strategies perfectly mirror the complex real-world ecological networks observed in our mNGS clinical cohort (Figure 1). The intensive care unit (ICU) and Department of Hyperbaric Oxygen Therapy exhibited particularly dense microbial–ARG co-occurrence networks, suggesting that these environments may represent hotspots of microbial interaction and ARGs exchange. In addition, patients receiving hyperbaric oxygen therapy in our hospital are frequently critically ill individuals with prolonged hospitalization histories and extensive prior antibiotic exposure. Such patients are often colonized or infected by multidrug-resistant organisms and may facilitate the convergence and dissemination of diverse ARGs within this specialized clinical setting. This clinical context may further contribute to the elevated ARG network complexity observed in the Department of Hyperbaric Oxygen Therapy and ICU. The exceptional density of resistance networks in the ICU and the Department of Hyperbaric Oxygen highlights how intense oxidative and antibiotic stress forces microbial communities to share ARGs (Martinez et al., 2023; Xu et al., 2025). Notably, the T6SS has been shown to mediate metal ion uptake (e.g., Mn^2+^, Zn^2+^) to combat reactive oxygen species (ROS) induced by antibiotics and hyperbaric environments (Si et al., 2017; Zhu et al., 2021; Liu et al., 2023; Li et al., 2024; Bhowmik et al., 2025). Thus, maintaining an active T6SS may provide physiological survival advantages in highly oxidative clinical niches, allowing complete lineages to persist, kill, and horizontally acquire resistance within complex polymicrobial communities(Si et al., 2017; Liaw et al., 2019; Yu et al., 2021).

While our study robustly links T6SS status to distinct resistome architectures and validates the bactericidal nature of the T6SS effectors *in vitro* (Figure 6), certain limitations remain. First, the proposed transformation-associated and plasmid-mediated acquisition models are inferred from genomic signatures rather than directly demonstrated through functional transfer experiments. Second, although T6SS status was consistently associated with resistome composition across multiple analytical frameworks, the magnitude of the observed effect was relatively small. This suggests that T6SS represents only one component of a broader ecological and evolutionary network shaping resistome diversification in *A. baumannii*. Future studies employing conjugation assays, transformation experiments, and polymicrobial infection models will be valuable for directly testing the evolutionary scenarios proposed here.

In summary, our findings demonstrate that T6SS status is consistently associated with distinct resistome and mobilome architectures in *A. baumannii*. T6SS-complete population were more frequently associated with endogenous and chromosomally linked resistance determinants, whereas T6SS-incomplete population were more commonly associated with acquired ARGs, expanded mobilomes, and mobile plasmid populations. *In vitro* functional assays further demonstrate that T6SS-positive strains possess a structurally and functionally active secretion system capable of mediating efficient interbacterial killing. Together, these patterns support the hypothesis that T6SS is associated with ecological and evolutionary differentiation of resistance gene acquisition strategies across clinical *A. baumannii* populations.

## Materials and methods

### Clinical data collection and co-occurrence analysis

We retrospectively collected mNGS reports positive for *Acinetobacter* spp. from The First Hospital of Jilin University between 2022 and 2025. A total of 873 samples were enrolled, including 738 samples positive for *A. baumannii*. Clinical metadata were extracted, comprising specimen sources, hospital departments, identified microbial pathogens, and ARGs. To investigate the co-occurrence patterns between microbial pathogens and ARGs, we constructed a binary presence-absence matrix. Features (pathogens or ARGs) detected in fewer than five samples were excluded to reduce data sparsity and ensure statistical robustness. Pairwise association analyses were evaluated using Fisher’s exact test, with *p*-values adjusted for multiple testing using the Benjamini–Hochberg false discovery rate (FDR) method. Statistically significant associations were defined by an FDR-adjusted *p* < 0.05 and an odds ratio (OR) > 1. The resulting co-occurrence network was constructed, visualized, and annotated using Cytoscape (v3.10.2) (Shannon et al., 2003).

### Genome retrieval and quality control

All publicly available *A. baumannii* whole-genome sequences were retrieved from the National Center for Biotechnology Information (NCBI, https://www.ncbi.nlm.nih.gov/) database on August 14, 2025. To ensure high-quality assemblies for downstream comparative genomics analysis, stringent quality control was performed using CheckM (v1.2.2) (Parks et al., 2015). Only genomes meeting the following criteria were retained: completeness > 97%, contamination < 3%, contig count < 50, and N50 value > 50,000 bp. Taxonomic accuracy was cross-verified using GTDB-Tk (v2.4.0)(Chaumeil et al., 2022); only genomes with consistent NCBI and GTDB-Tk classifications were included (*n* = 2,946). To enhance annotation consistency, genome annotation was performed using Prokka (Seemann, 2014) supplemented with a custom protein database derived from the *A. baumannii* ATCC 17978 genome (accession: GCA_902728005.1).

### Identification and classification of T6SS

The core components of the T6SS were identified using Proteinortho (v6.0.29) (Lechner et al., 2011) against the SecReT6 (Zhang et al., 2023) database. The T6SS in *A. baumannii* consists of 12 core components (*tssA*–*tssM*), lacking the *tssJ* lipoprotein typically present in most Gram-negative bacteria (Carruthers et al., 2013; Kandolo et al., 2023). To ensure a high-fidelity classification, we integrated gene content and locus organization information. Genomes were categorized into three groups: complete T6SS (*n* = 2,480): Genomes harboring all 12 core components, with 11 components (*tssA*–*tssH* and *tssK*–*tssM*) organized in a continuous genomic locus (intervening genes ≤5)(Dong et al., 2025; Zhu et al., 2025). Given its variable genomic localization, the *tssI* gene was required to be present but not necessarily syntenic with the main cluster. Incomplete T6SS (*n* = 435): Genomes were defined as: (i) a continuous locus containing ≤ 10 core components; or (ii) harboring 11 core components where the missing gene was located within an internal genomic region of a contig, confirming a true genomic deletion. Ambiguous T6SS (*n* = 31, excluded): Genomes harboring 11 core components where the missing gene was located at a contig boundary. These cases were treated as potential assembly artifacts and were excluded from subsequent analysis to prevent false-positive identification.

### ST typing and phylogenetic tree construction

Sequence types (STs) were determined using the MLST (v2.23.0) (Jolley et al., 2018) Pasteur scheme for *A. baumannii*. Highly conserved marker genes extracted from the 2,915 *A. baumannii* genomes were aligned using PhyloPhlAn (v3.0.67) (Asnicar et al., 2020). Subsequently, a maximum-likelihood (ML) tree was inferred using IQ-TREE (v2.1.4) (Minh et al., 2020) with the best-fit substitution model (VT + R10). Branch support was assessed using 10,000 ultrafast bootstrap replicates, while other parameters were set to default. The resulting tree was rooted using *Acinetobacter pittii* as the outgroup and visualized using iTOL (v7.1, https://itol.embl.de/) (Letunic and Bork, 2024).

### Identification of ARGs, MGEs, and plasmids

ARGs were identified using the Resistance Gene Identifier (RGI, v 6.0.3) against the Comprehensive Antibiotic Resistance Database (CARD) (Alcock et al., 2023), retaining “perfect” and “strict” hits to ensure high confidence. MGEs were identified using MobileElementFinder (v1.1.2) (Durrant et al., 2020) with default parameters, which mainly comprised ISs, Tns, and composite transposons. Plasmids were detected at the contig level using geNomad (v1.11.1) (Camargo et al., 2024) and further classified using MOB-suite (v 1.4.9) (Robertson et al., 2020) to predict their mobility. An ARG was classified as “plasmid-borne” if its genomic location was fully contained within a contig identified as a plasmid; otherwise, it was considered “chromosome-borne.”

### ARG burden analysis

To quantify the ARG burden, we employed three complementary metrics: (1) total ARG count per genome, (2) total carbapenemase gene count, and (3) carbapenemase gene richness (unique carbapenemase gene types). Given that the data distributions deviated from normality (assessed via the Shapiro–Wilk test), a Mann–Whitney test was performed to compare the ARG burden between groups. To robustly estimate the magnitude of differences, we calculated Cliff’s delta and determined 95% confidence intervals (CIs) for median differences using bootstrap resampling (*n* = 5,000 iterations).

### Diversity and differential analysis of ARGs

Alpha diversity of ARGs was assessed at the genome level using ARG richness (non-redundant ARG) and the Shannon diversity index based on presence/absence matrix of ARGs, comparing groups via the Wilcoxon rank-sum test. To further account for phylogenetic non-independence and genome-level covariates, PGLS regression was performed incorporating phylogenetic covariance, genome size, and assembly fragmentation metrics. Beta diversity was calculated based on the Bray-Curtis’ dissimilarity matrices calculated from ARG abundance profiles. Statistical significance of group separation and homogeneity of multivariate dispersion were assessed using PERMANOVA and PERMDISP (999 permutations), respectively. Resistome composition was visualized using PCoA.

To control for potential confounding by clonal population structure, lineage-aware analyses were additionally performed using sequence type (ST) as a permutation constraint in stratified PERMANOVA. Analyses were restricted to STs containing both T6SS-complete and T6SS-incomplete genomes to ensure informative permutations. Sensitivity analyses were further performed by excluding the dominant ST2 lineage, and by independently evaluating major lineages (ST2 and ST499) to assess the robustness of observed resistome patterns.

Furthermore, Differential enrichment analyses of individual ARGs between T6SS groups were performed using Fisher’s exact test based on ARG presence/absence profiles. *p*-values were adjusted for multiple comparisons using the Benjamini–Hochberg FDR method. ARGs with an adjusted *p* < 0.05 and an absolute log2 odds ratio (|log2OR|) > 1 were considered significantly enriched. The consistency of these enrichment signals was further verified across multiple ST backgrounds and after excluding the dominant ST2 lineage.

### Phylogenetically informed regression analysis (PGLS)

To account for evolutionary non-independence among genomes, phylogenetic generalized least squares (PGLS) regression was performed using the R package caper (v1.0.4) (Orme et al., 2013). The phylogenetic tree, rooted with *Acinetobacter pittii*, was pruned to retain genomes analyzed in this study (*n* = 2,915), from which a variance–covariance matrix was derived. Separate PGLS models were constructed for ARG abundance, ARG richness, and Shannon diversity index. In each model, T6SS status was treated as a binary categorical predictor, while genome size, and assembly fragmentation (log-transformed contig count) were included as covariates after standardization. Pagel’s *λ* was estimated by maximum likelihood to account for phylogenetic signal and evolutionary non-independence among genomes.

### Rarefaction analysis of resistome and mobilome diversity

To evaluate and compare the cumulative diversity of ARGs and MGEs across populations, we performed rarefaction analyses. Sample sizes were normalized to avoid bias from group imbalance. Specifically, the larger T6SS-complete population (*n* = 2,480) was randomly downsampled without replacement to match the smaller population (*n* = 435). This resampling procedure was repeated 1,000 times to account for stochastic sampling effects. Rarefaction curves were constructed by plotting the mean richness at each sampling depth, with 95% confidence intervals estimated from the resampling distribution to quantify uncertainty.

### Correlation analysis between ARGs and MGEs abundance

The association between the abundance of ARGs and MGEs at the genome level was evaluated using Spearman’s rank correlation coefficient (R). Correlation coefficients and corresponding *p*-values were calculated based on two-sided tests, and *p*-value < 0.05 was considered to indicate a significant correlation. Correlation coefficients were calculated using the SciPy (v1.15.3) library, while all corresponding data visualizations were generated using Matplotlib (v3.8.4) and Seaborn (v0.13.2) in a Python (v3.11.5) environment.

### Proximity analysis of ARGs and MGEs

To evaluate the mobilization potential of ARGs, we calculated the genomic distance between each ARGs and its nearest downstream or upstream MGEs located on the same contig. To ensure the stringency of potential functional associations and minimize false positives, only ARG-MGE pairs within a 5 kb window were retained for downstream analysis. This distance threshold was selected based on established criteria for inferring functional genetic linkage in ARG–MGE co-localization studies(Che et al., 2021; Maciel-Guerra et al., 2023). The physical distance was defined as the number of base pairs (bp) between the nearest boundaries of the two features. The distributions of these genomic distances were subsequently compared between groups by the Wilcoxon rank-sum test.

### Identification of VgrG and its downstream effector proteins across AB308 strains

To systematically identify candidate T6SS effectors, we implemented a VgrG-centered genome mining pipeline. VgrG homologs were identified using HMMER (v3.2) (Potter et al., 2018) with an E-value < 1e−6, serving as anchor genes for T6SS effectors. For each identified *vgrG* gene, protein-coding genes located immediately downstream on the same strand and within the same contig were extracted. These genes were considered putative effector candidates and its cognate immunity protein based on their genomic co-localization with *vgrG* (Jiang et al., 2025). These downstream proteins were aligned against the NCBI non-redundant (nr) protein database using BLASTp to retrieve preliminary functional annotations. To securely differentiate bona fide effectors from non-toxic passenger proteins, candidates underwent rigorous conserved domain analysis via NCBI’s Conserved Domain Database (CDD). Presumptive T6SS effectors were shortlisted based on the presence of typical polymorphic toxin domains, genomic orientation, and literature evidence. The architecture of these identified genetic loci were subsequently visualized using ChiPlot (https://www.chiplot.online/), and candidate effectors were subsequently validated via empirical toxicity assays.

### Construction of effector protein expression vectors

To evaluate the *in vitro* toxicity and expression of the six putative T6SS effectors (E1–E6) identified in *A. baumannii* strain AB308, the corresponding coding sequences were amplified and cloned into inducible expression vectors. Genomic DNA from strain AB308 was extracted using the TIANamp Bacteria DNA Kit (Tiangen biochemical technology, Beijing, China) and served as the template. The full-length open reading frames of E1–E6 were amplified using high-fidelity DNA polymerase (TransGen Biotech, Beijing, China) with specific primer pairs containing appropriate restriction enzyme sites. For IPTG-inducible expression, the amplified fragments were digested and ligated into the pTac vector downstream of the tac promoter. For arabinose-inducible expression, the fragments were cloned into the pJL03/pJL01 vector under the control of the pBAD promoter. The recombinant plasmids, alongside their respective empty vectors, were initially transformed into *E. coli* DH5α for downstream toxicity and protein expression assays. The identity and sequence accuracy of all constructs were verified by colony PCR and Sanger sequencing to ensure correct orientation and reading frame. Transformants were selected on Luria-Bertani (LB) agar plates supplemented with appropriate antibiotics (gentamicin 10 μg/mL or kanamycin 30 μg/mL).

### Bacterial viability and spotting assays

The cellular toxicity of the putative T6SS effectors was assessed using spotting viability assays in *E. coli* DH5α. Effector genes were expressed individually in the absence of their cognate immunity genes. Overnight cultures were normalized to an initial OD_600_ of 1.0 and serially diluted 5-fold in sterile phosphate-buffered saline (PBS, pH 7.4). A 5 μL aliquot of each dilution was spotted onto LB agar plates containing appropriate antibiotics. For induction, the plates were supplemented with either 0.2 mM IPTG (for pTac constructs) or 1% (w/v) L-arabinose (for pJL03 constructs). Plates lacking the inducers served as non-inducing controls. The plates were incubated at 37 °C for 16–18 h, after which bacterial viability was assessed based on growth patterns across serial dilutions and documented by photography.

### Interbacterial competition assay

Interspecies bacterial competition assays were performed to evaluate the T6SS-dependent predatory activity of *A. baumannii* against *E. coli* (Luo et al., 2023; Yang et al., 2025). Wild-type *A. baumannii* and the Δ*tssM* mutant, together with *E. coli* DH5α as prey, were grown overnight in appropriate liquid media. Cells were harvested by centrifugation, washed three times with sterile PBS, and resuspended to adjust the OD_600_nm to 1.0. The predator and prey suspensions were mixed at a predetermined ratio. Aliquots (5 μL) of the mixtures were spotted onto dry, antibiotic-free LB agar plates and incubated at 37 °C for 5 h to allow interbacterial killing. The co-culture spots were then excised, resuspended in 1 mL of sterile PBS, and vigorously vortexed to release the bacteria. The target survival kinetics were verified and recorded via spotting serial ten-fold dilutions onto selective agar plates containing appropriate antibiotics to exclusively support prey growth, followed by incubation overnight at 37 °C.

### Bacterial growth curve and toxicity quantification

For liquid toxicity validation assays (Sun et al., 2024), overnight cultures of *E. coli* harboring the respective expression constructs were diluted 1:100 into fresh LB broth containing appropriate antibiotics. The cultures were incubated at 37 °C with shaking at 220 rpm until the optical density at 600 nm reached approximately 0.4–0.6. To induce the expression of candidate effectors, IPTG was added to a final concentration of 0.2 mM, while cultures without IPTG served as uninduced controls. The OD_600_nm values were measured automatically every hour over a 12 h course using a microplate reader (BioTek, USA) with continuous shaking. All assays were conducted with three independent biological replicates, each containing three technical replicates.

### Total protein SDS-PAGE and Coomassie Brilliant Blue staining

To verify equal protein loading across all lanes, total cellular proteins were resolved on a 12% SDS-PAGE gel. Following electrophoresis, the gel was immersed in Coomassie Brilliant Blue R-250 staining solution [0.1% (w/v) Coomassie R-250, 45% (v/v) methanol, and 10% (v/v) glacial acetic acid] and incubated at room temperature for 1 h with gentle shaking. The gel was subsequently transferred to a destaining solution [40% (v/v) methanol and 10% (v/v) glacial acetic acid] and agitated until the background became completely clear and protein bands were distinctly visualized. The destained gel was imaged using a gel documentation system (Bio-Rad ChemiDoc) to evaluate sample loading uniformity.

### Protein expression analysis by Western blot

To verify the expression of the six candidate T6SS effectors (E1–E6) in the heterologous *E. coli* host, Western blot analysis was performed. *E. coli* strains harboring corresponding expression plasmids were cultured in LB medium to an OD_600_nm of approximately 0.5. Cultures were divided into two subcultures: one was induced with 0.2 mM IPTG or 1% L-arabinose, and the other was left uninduced as a negative control. After induction for 6 h (IPTG) or overnight (L-arabinose) at 37 °C, equivalent amounts of bacterial cells were harvested by centrifugation.

The cell pellets were resuspended in 1 × SDS-PAGE loading buffer, boiled at 95 °C for 10 min, and the lysates were separated on 10% SDS-polyacrylamide gels. Proteins were subsequently transferred onto polyvinylidene difluoride (PVDF) membranes (Millipore, USA). The membranes were blocked with 5% non-fat milk in Tris-buffered saline containing 0.1% Tween 20 (TBST) for 1 h at room temperature. For immunodetection, the expressed effector proteins were fused with a C-terminal 6 x His-tag and detected via Immunoblotting (IB) using an anti-His antibody. The membranes were probed overnight at 4 °C with mouse anti-His tag-corresponding primary antibodies (Proteintech, USA). After extensive washing with TBST, the membranes were incubated with horseradish peroxidase (HRP)-conjugated secondary antibodies (Proteintech, USA) for 1 h at room temperature. The protein bands were visualized using an enhanced chemiluminescence (ECL) detection system (Tanon, Shanghai, China) and imaged with a gel documentation system (Bio-Rad ChemiDoc).

## Data Availability

All datasets analyzed in this study are publicly available from the NCBI database. Detailed accession numbers and metadata for all samples are provided in Supplementary Table S2. The genome of AB308 has been deposited in the European Nucleotide Archive (ENA) database under BioProject accession PRJEB113810 and BioSample SAMEA122731147.

## Supplementary material

SUPPLEMENTARY FIGURE 1 Comparative analysis of carbapenemase genes and overall resistome composition between T6SS-complete and T6SS-incomplete groups.**(A–B)** Genomic carbapenemase genes characterized by **(A)** total abundance and **(B)** richness (number of unique carbapenemase genes). Violin plots illustrate data distribution, with internal boxplots representing the median and interquartile range (IQR). Statistical significance was determined by the Wilcoxon rank-sum test. **(C)** Multivariate dispersion (PERMDISP) analysis of the overall resistome composition based on Bray–Curtis distances. Each point represents the distance of an individual genome to the group centroid, and boxplots indicate the median and interquartile range (IQR).

SUPPLEMENTARY FIGURE 2 Distribution of enriched ARGs and sensitivity analysis excluding the dominant ST2 lineage. **(A)** Sankey diagram illustrating the distribution of significantly enriched ARGs across T6SS status and ST types. **(B)** Sensitivity analysis of ARG composition in the non-ST2 population. Beta diversity was visualized using Bray–Curtis-based PCoA, with group separation and dispersion homogeneity evaluated by PERMANOVA and PERMDISP, respectively. **(C)** Differential ARG enrichment identified using Fisher’s exact test. Ellipses in the PCOA plot represent 95% confidence intervals.

SUPPLEMENTARY FIGURE 3 Lineage-stratified analyses of T6SS-associated resistome differentiation. **(A)** ARG composition analysis within ST2 lineage. **(B)** ARG composition analysis within ST499 lineage. For both, beta diversity was visualized using Bray–Curtis-based PCoA, with group separation and dispersion homogeneity evaluated by PERMANOVA and PERMDISP, respectively. Differentially enriched ARGs in ST2 are shown in the accompanying volcano plot. Ellipses represent 95% confidence intervals.

SUPPLEMENTARY FIGURE 4 T6SS-mediated interbacterial antagonism by *A. baumannii* AB308. **(A)** Killing of *E. coli* prey by indicated *A. baumannii* strains (WT or Δt*ssM*). Predators and prey were mixed at an initial OD_600_nm of 1.0. Left: Spot assay using 5-fold serial dilutions. Right: Quantitative CFU determination of surviving prey after co-culture. **(B)** 12-hour growth dynamics of *E. coli* prey during co-culture with WT or Δ*tssM A. baumannii* strains. **(C)** Western blot of Hcp expression (~17 kDa) in culture supernatants (secreted) and cell lysate (cellular) of indicated strains. Statistical significance was determined using a one-way ANOVA followed by Dunnett's multiple comparisons test (**, *p* < 0.01; ns, not significant)

SUPPLEMENTARY Figure 5 Intracellular toxicity profiles of the six candidate T6SS effectors in *E. coli*. Growth dynamics of *E. coli* strains heterologously expressing individual candidate effectors (E1–E6) or carrying the empty vector (control) over a 12-hour cultivation period post-induction. The specific expression vectors (pTac, pJL01-pelB, or pJL03) utilized for each effector are indicated. Data represent the mean ± standard deviation (SD) from three independent biological replicates (*n* = 3).

SUPPLEMENTARY FIGURE 6 CBB-stained SDS-PAGE of whole-cell lysates from *E. coli* expressing effectors E3 or E4. Lysates from empty vector (pJL01-pelB), pJL01-pelB-E3, or pJL03-E4 were analyzed under uninduced (−) or arabinose-induced (+) conditions. Protein markers (kDa) are shown on the left. The arrows indicate the predicted molecular weight.
