# Supplementary figures and images for: Type VI secretion system completeness shapes evolutionary trade-offs in the Acinetobacter baumannii resistome
Source: Front Microbiol. 2026 Jul 15;17:1867466. doi: 10.3389/fmicb.2026.1867466 (PMC13416068; doi:10.3389/fmicb.2026.1867466)

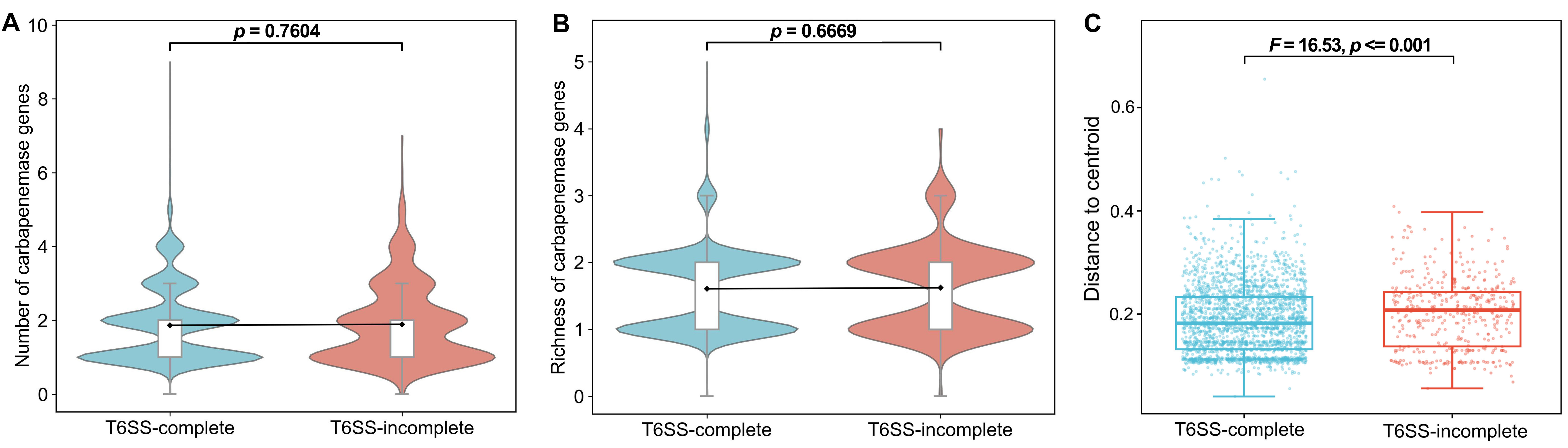

Supplement: SUPPLEMENTARY FIGURE 1 — Comparative analysis of carbapenemase genes and overall resistome composition between T6SS-complete and T6SS-incomplete groups.(A–B) Genomic carbapenemase genes characterized by (A) total abundance and (B) richness (number of unique carbapenemase genes). Violin plots illustrate data distribution, with internal boxplots representing the median and interquartile range (IQR). Statistical significance was determined by the Wilcoxon rank-sum test. (C) Multivariate dispersion (PERMDISP) analysis of the overall resistome composition based on Bray–Curtis distances. Each point represents the distance of an individual genome to the group centroid, and boxplots indicate the median and interquartile range (IQR). [file Image_1.JPEG]

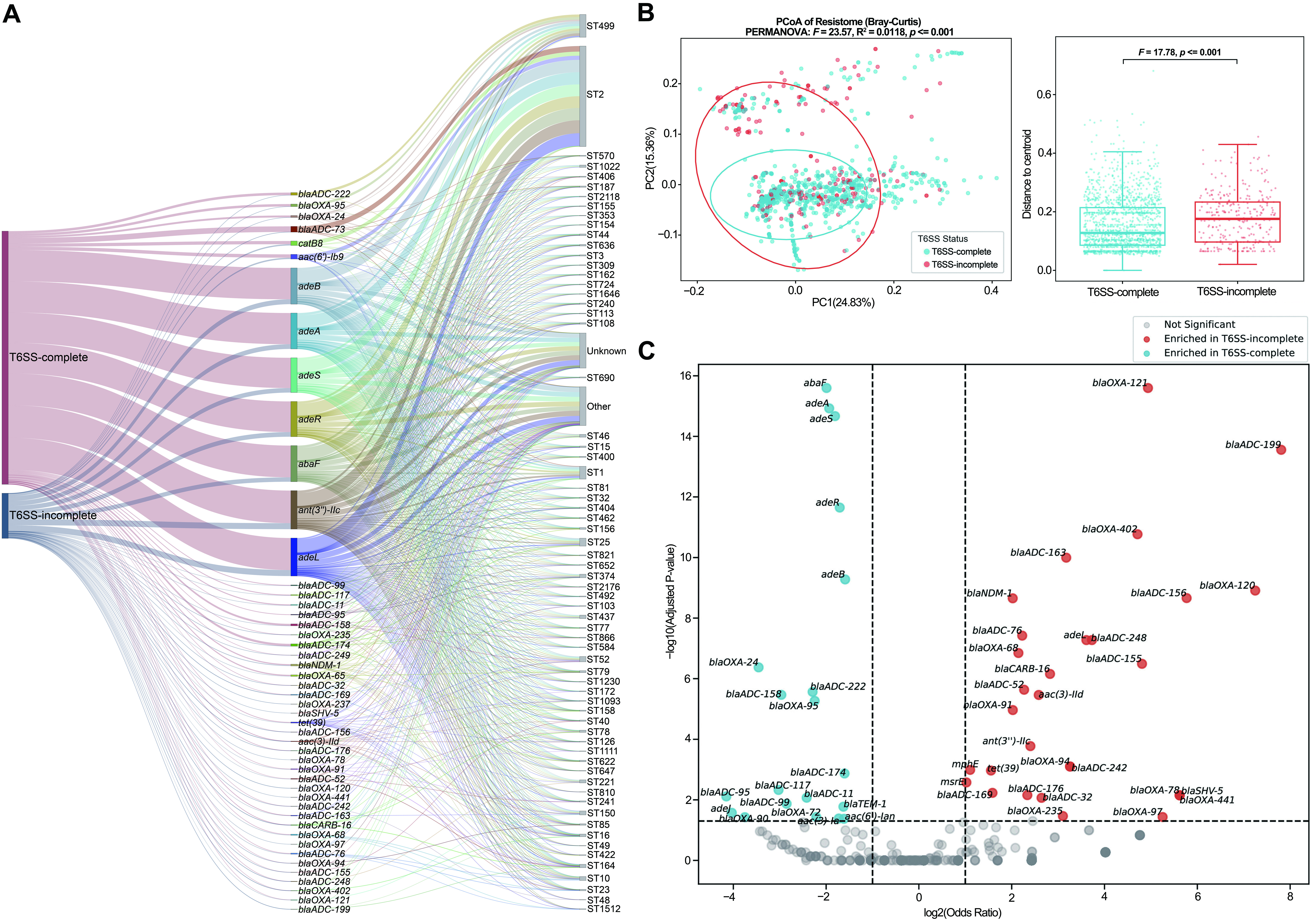

Supplement: SUPPLEMENTARY FIGURE 2 — Distribution of enriched ARGs and sensitivity analysis excluding the dominant ST2 lineage. (A) Sankey diagram illustrating the distribution of significantly enriched ARGs across T6SS status and ST types. (B) Sensitivity analysis of ARG composition in the non-ST2 population. Beta diversity was visualized using Bray–Curtis-based PCoA, with group separation and dispersion homogeneity evaluated by PERMANOVA and PERMDISP, respectively. (C) Differential ARG enrichment identified using Fisher’s exact test. Ellipses in the PCOA plot represent 95% confidence intervals. [file Image_2.JPEG]

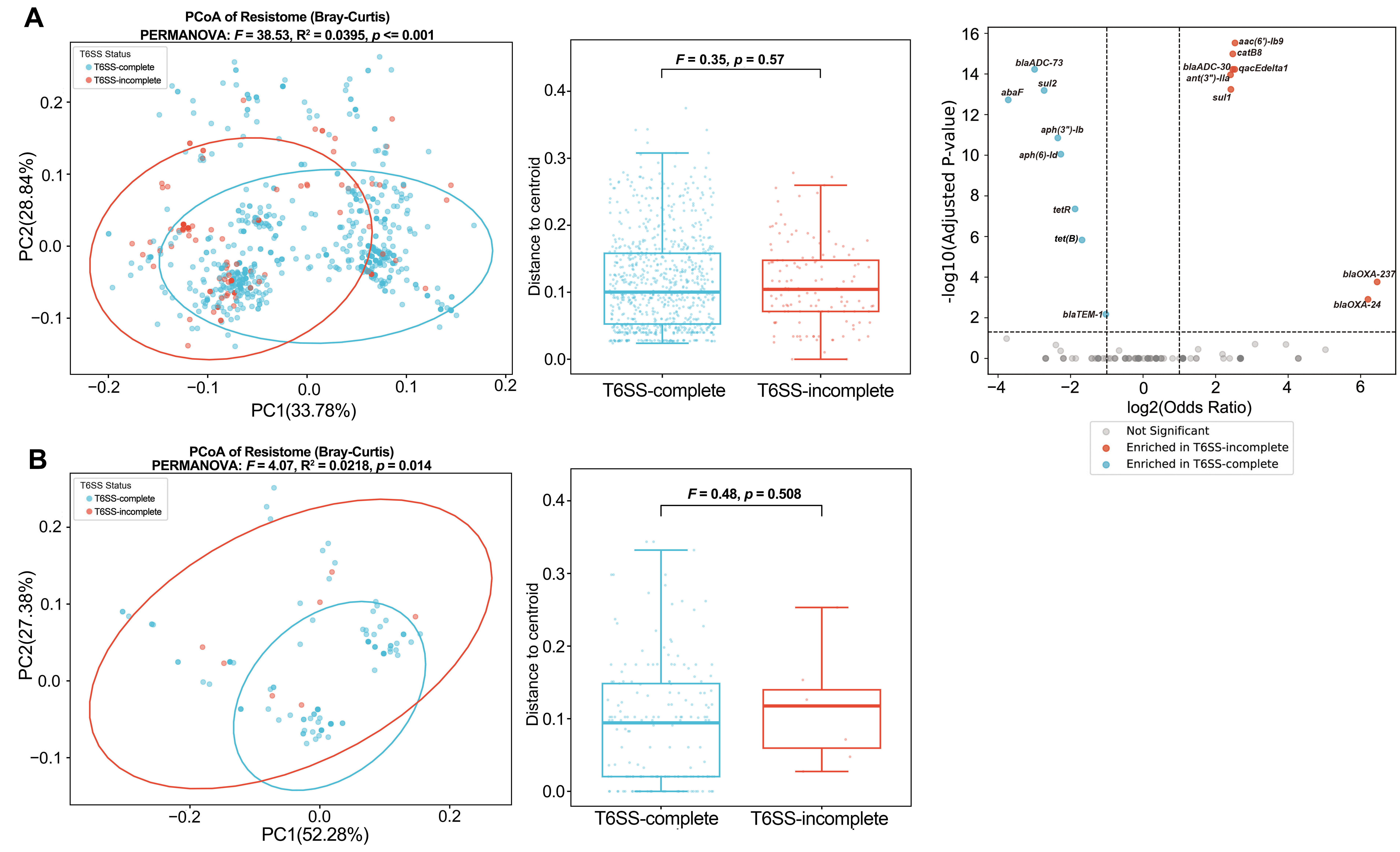

Supplement: SUPPLEMENTARY FIGURE 3 — Lineage-stratified analyses of T6SS-associated resistome differentiation. (A) ARG composition analysis within ST2 lineage. (B) ARG composition analysis within ST499 lineage. For both, beta diversity was visualized using Bray–Curtis-based PCoA, with group separation and dispersion homogeneity evaluated by PERMANOVA and PERMDISP, respectively. Differentially enriched ARGs in ST2 are shown in the accompanying volcano plot. Ellipses represent 95% confidence intervals. [file Image_3.JPEG]

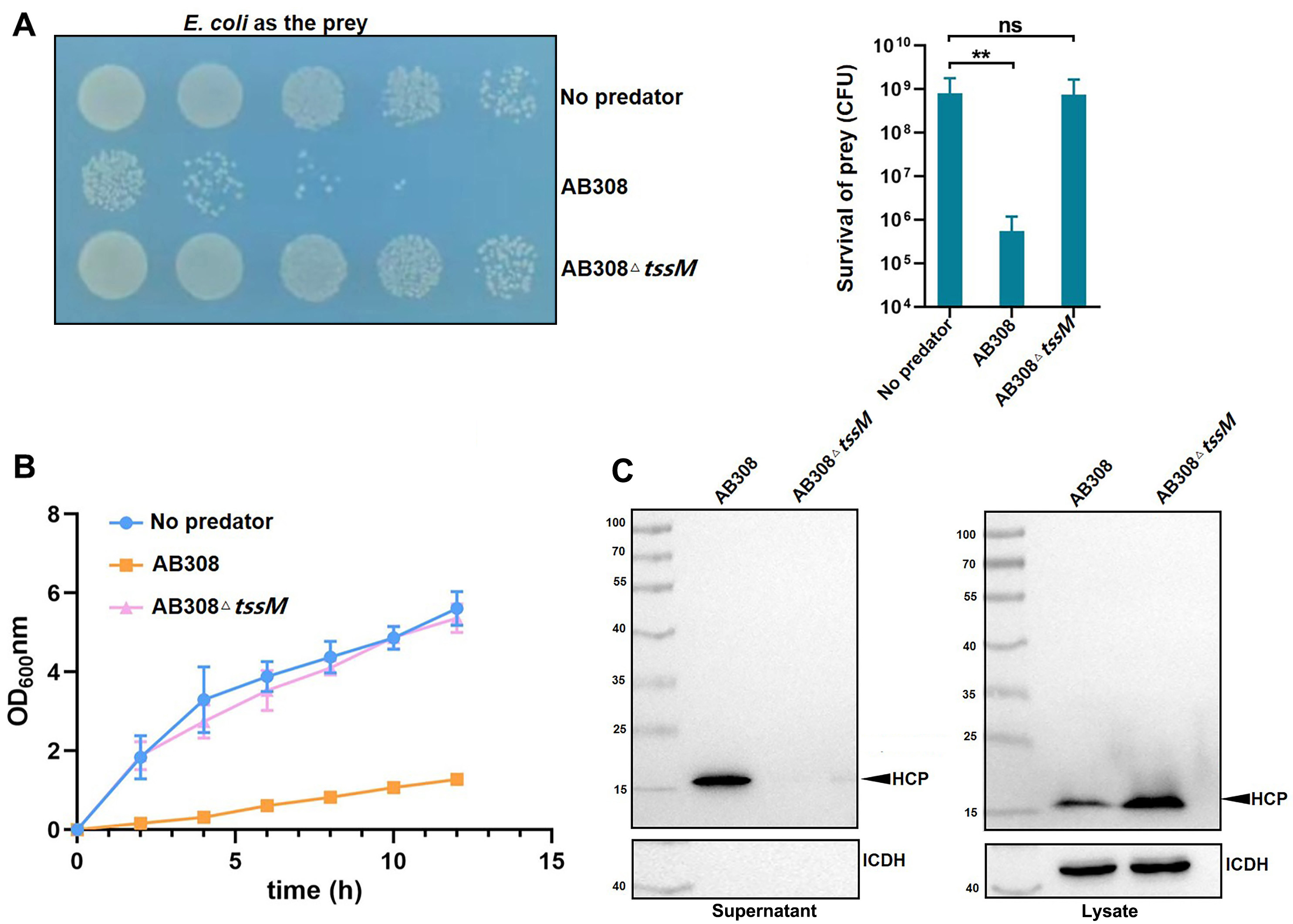

Supplement: SUPPLEMENTARY FIGURE 4 — T6SS-mediated interbacterial antagonism by A. baumannii AB308. (A) Killing of E. coli prey by indicated A. baumannii strains (WT or ΔtssM). Predators and prey were mixed at an initial OD600nm of 1.0. Left: Spot assay using 5-fold serial dilutions. Right: Quantitative CFU determination of surviving prey after co-culture. (B) 12-hour growth dynamics of E. coli prey during co-culture with WT or ΔtssM A. baumannii strains. (C) Western blot of Hcp expression (~17 kDa) in culture supernatants (secreted) and cell lysate (cellular) of indicated strains. Statistical significance was determined using a one-way ANOVA followed by Dunnett's multiple comparisons test (**, p < 0.01; ns, not significant) [file Image_4.JPEG]

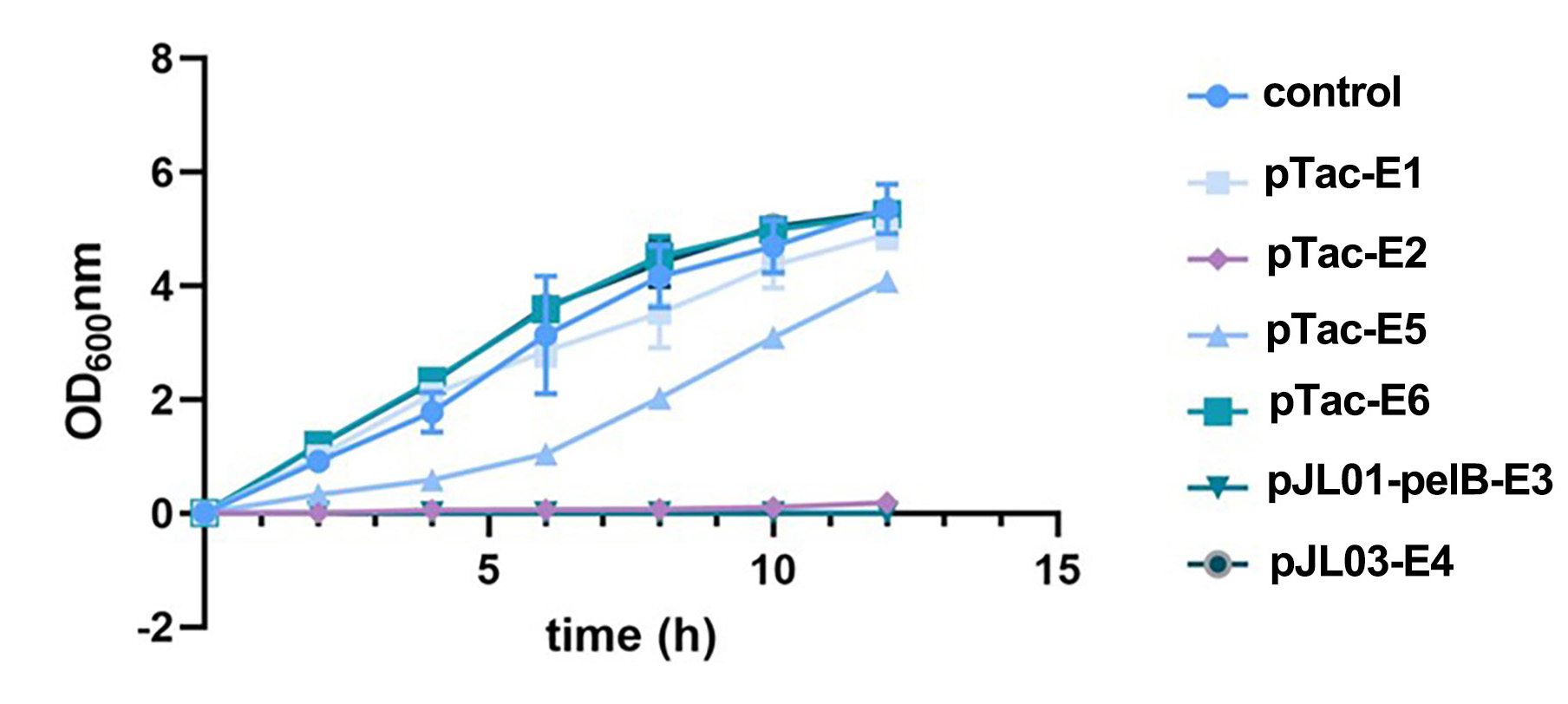

Supplement: SUPPLEMENTARY Figure 5 — Intracellular toxicity profiles of the six candidate T6SS effectors in E. coli. Growth dynamics of E. coli strains heterologously expressing individual candidate effectors (E1–E6) or carrying the empty vector (control) over a 12-hour cultivation period post-induction. The specific expression vectors (pTac, pJL01-pelB, or pJL03) utilized for each effector are indicated. Data represent the mean ± standard deviation (SD) from three independent biological replicates (n = 3). [file Image_5.JPEG]

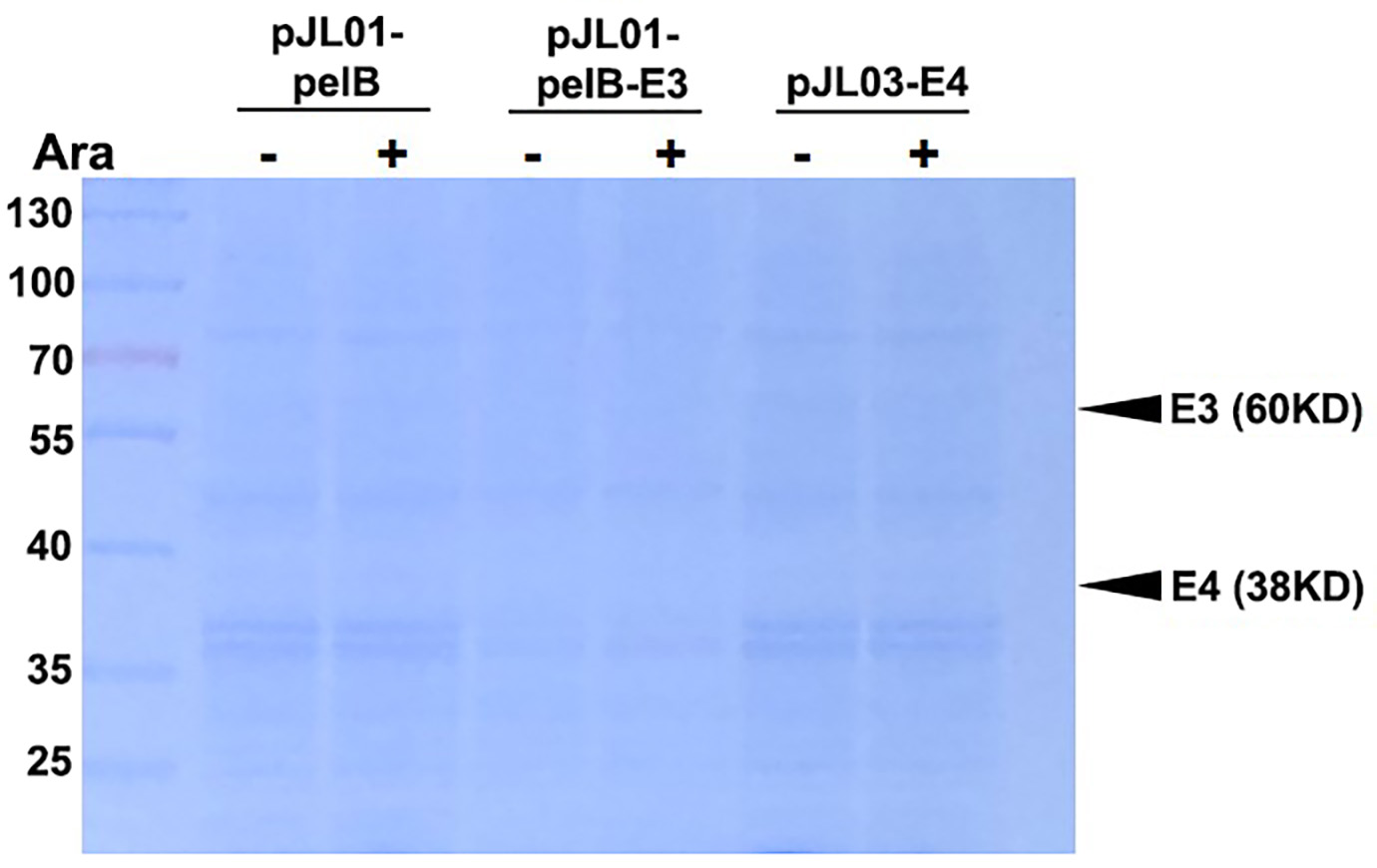

Supplement: SUPPLEMENTARY FIGURE 6 — CBB-stained SDS-PAGE of whole-cell lysates from E. coli expressing effectors E3 or E4. Lysates from empty vector (pJL01-pelB), pJL01-pelB-E3, or pJL03-E4 were analyzed under uninduced (−) or arabinose-induced (+) conditions. Protein markers (kDa) are shown on the left. The arrows indicate the predicted molecular weight. [file Image_6.JPEG]

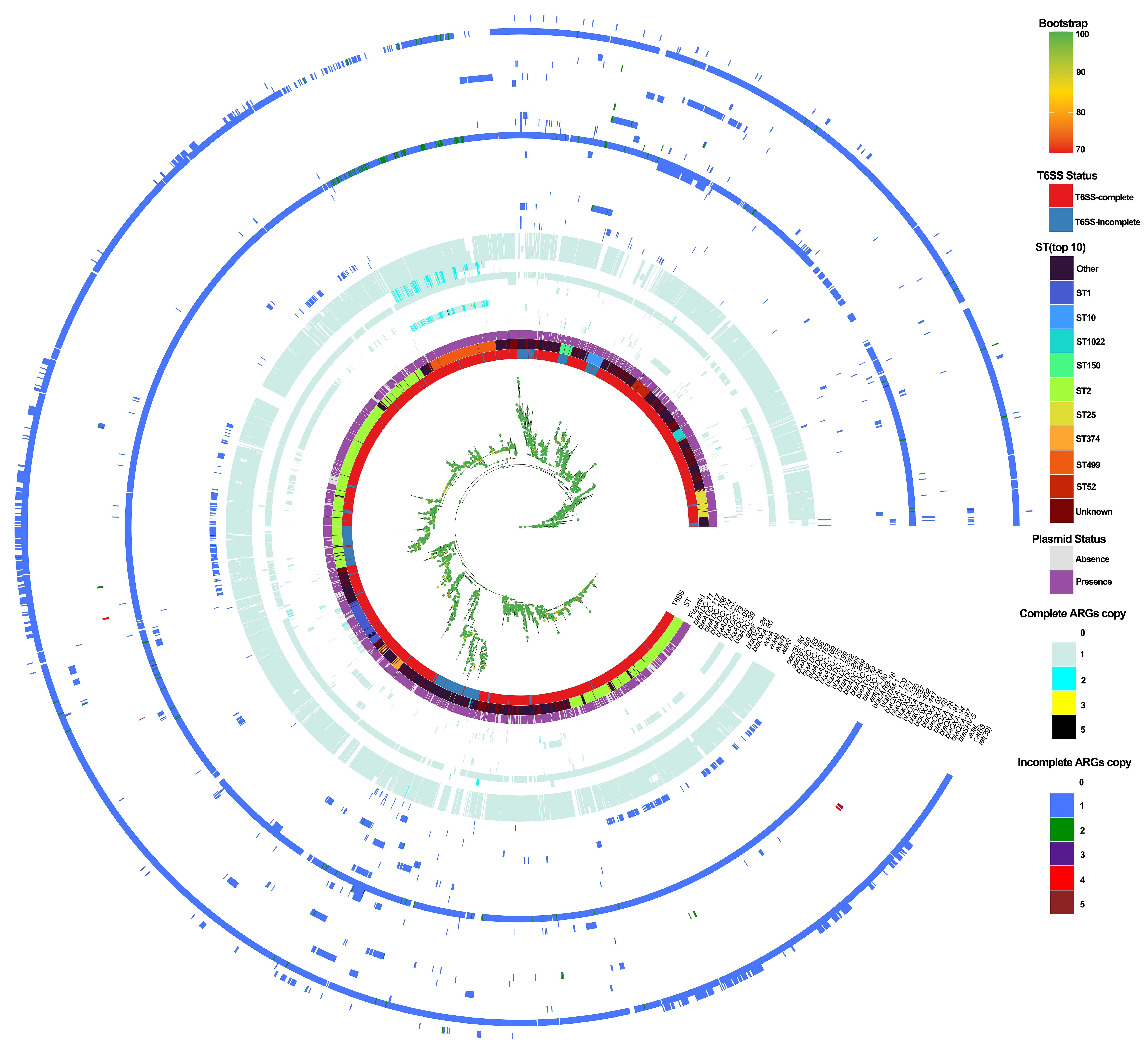

Supplement: Supplementary file 7 [file Data_Sheet_1.PDF]
