# Supplementary material for: Type VI secretion system completeness shapes evolutionary trade-offs in the Acinetobacter baumannii resistome
Source: Front Microbiol. 2026 Jul 15;17:1867466. doi: 10.3389/fmicb.2026.1867466 (PMC13416068; doi:10.3389/fmicb.2026.1867466)

biological and technical replicates 2，Figure 6B

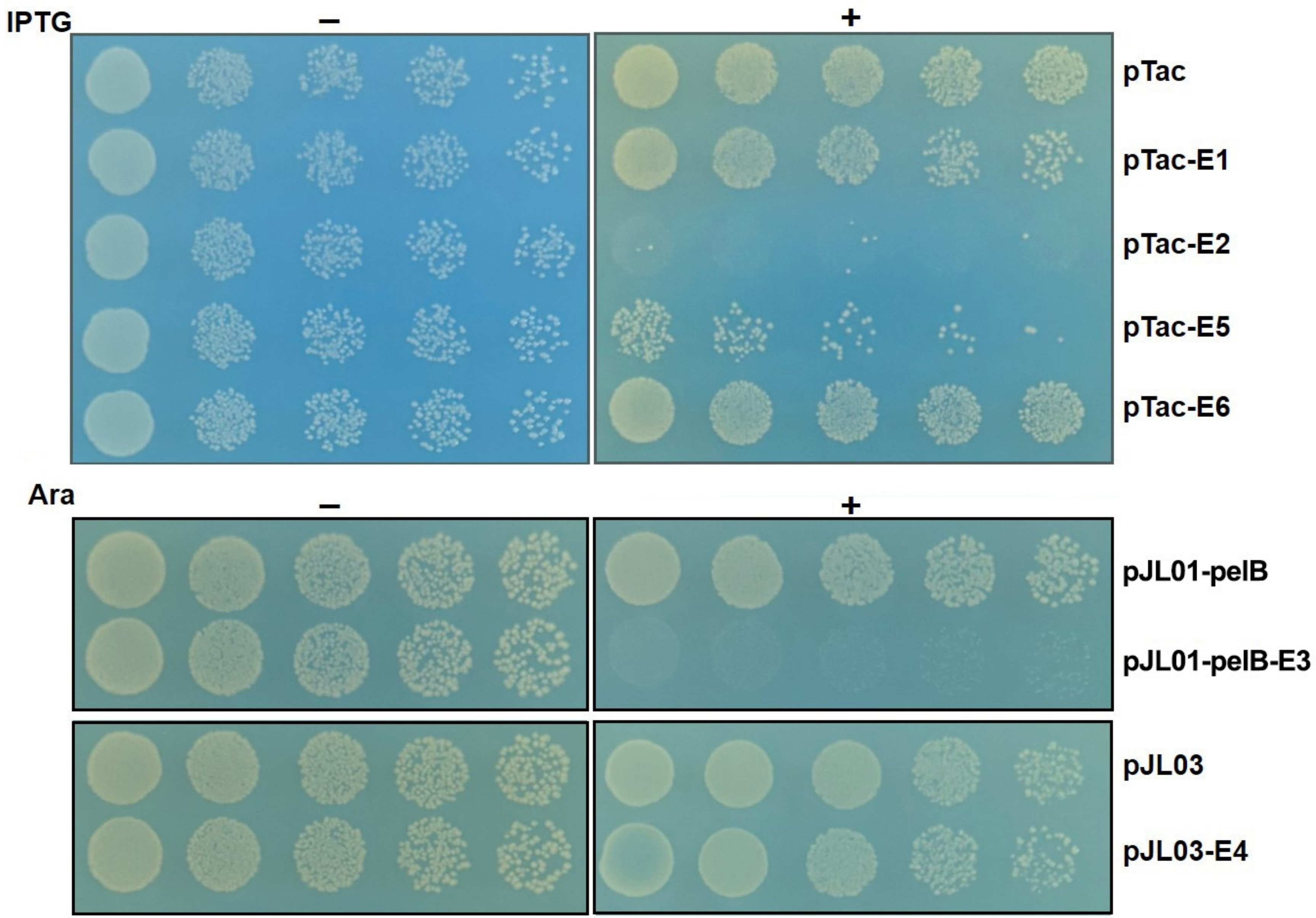

biological and technical replicates 3，Figure 6B

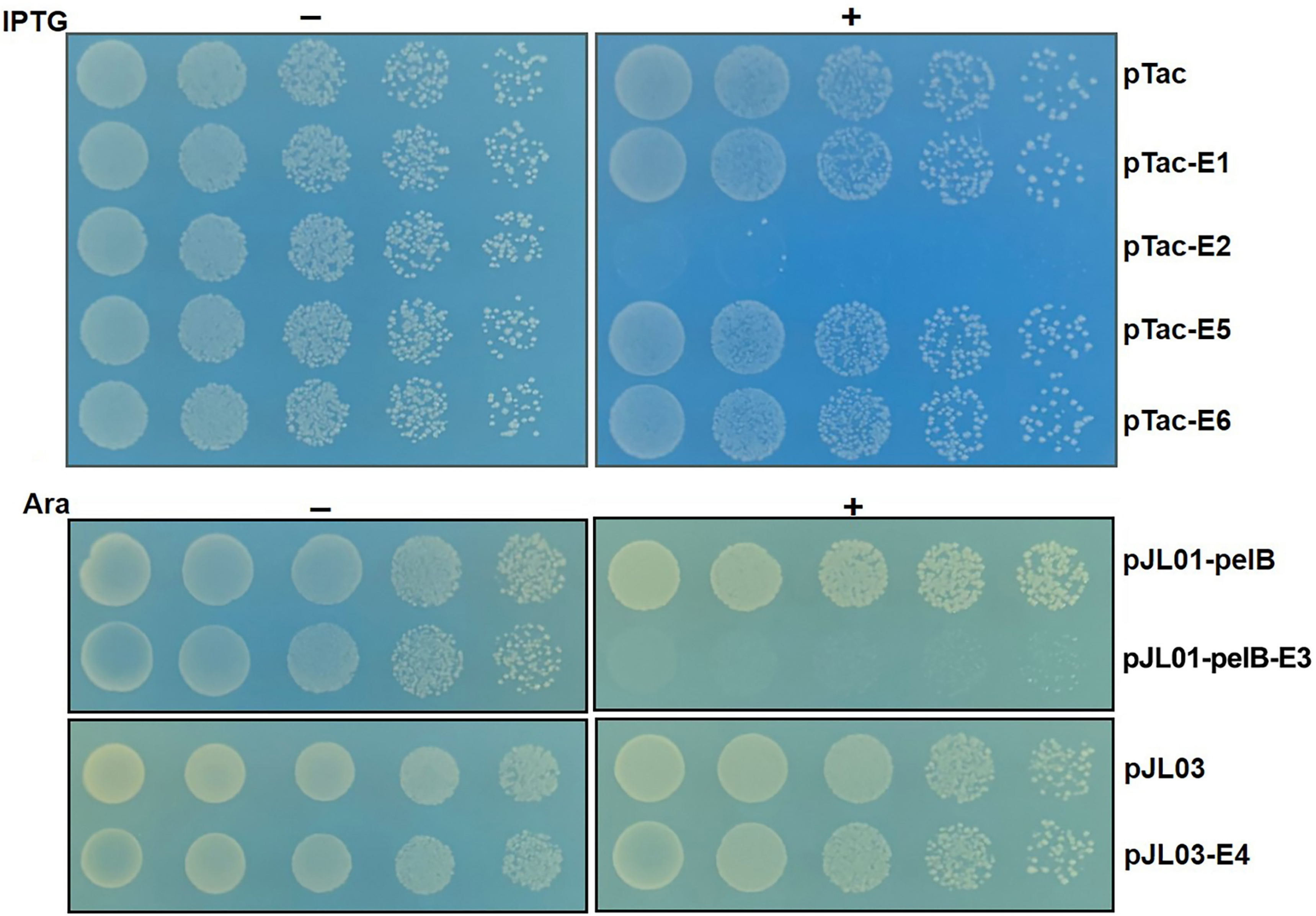

biological and technical replicates 2，Figure 6C

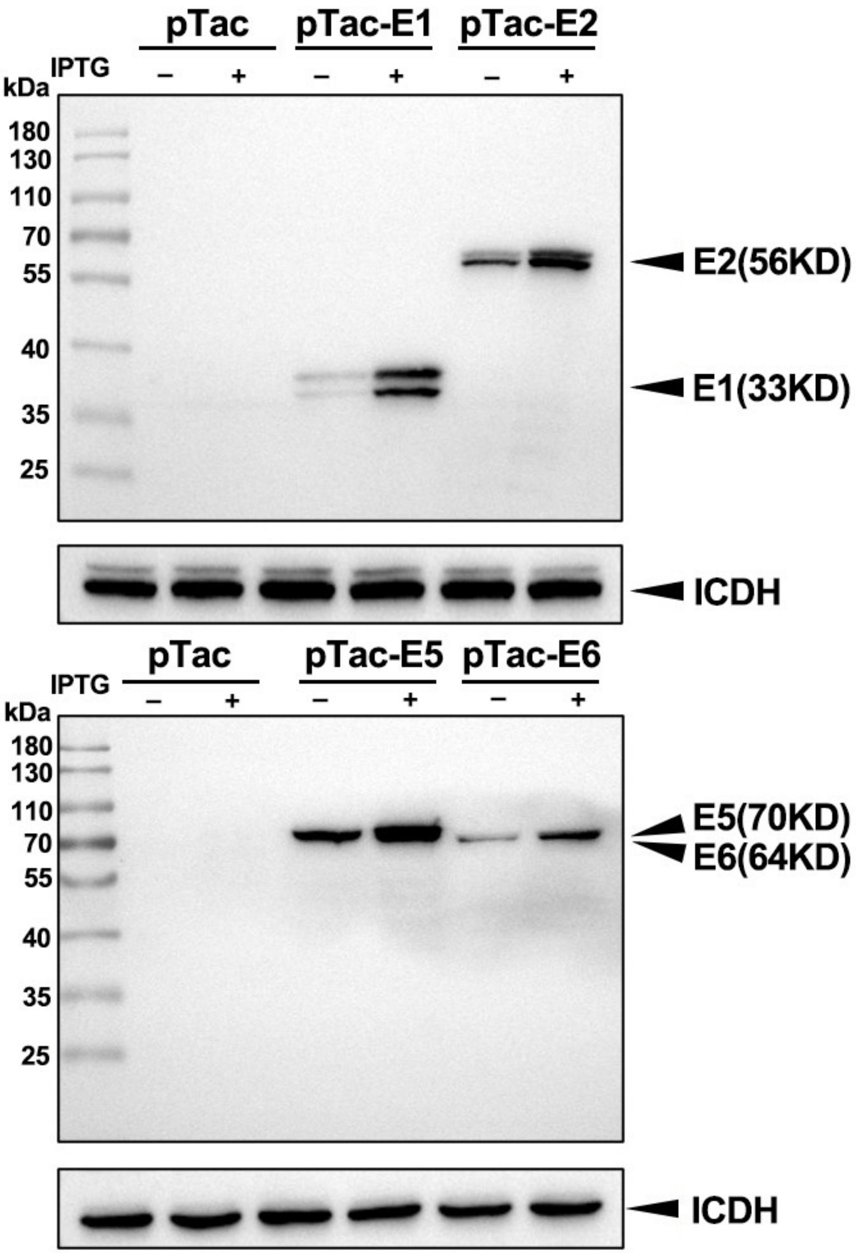

biological and technical replicates 3，Figure 6C

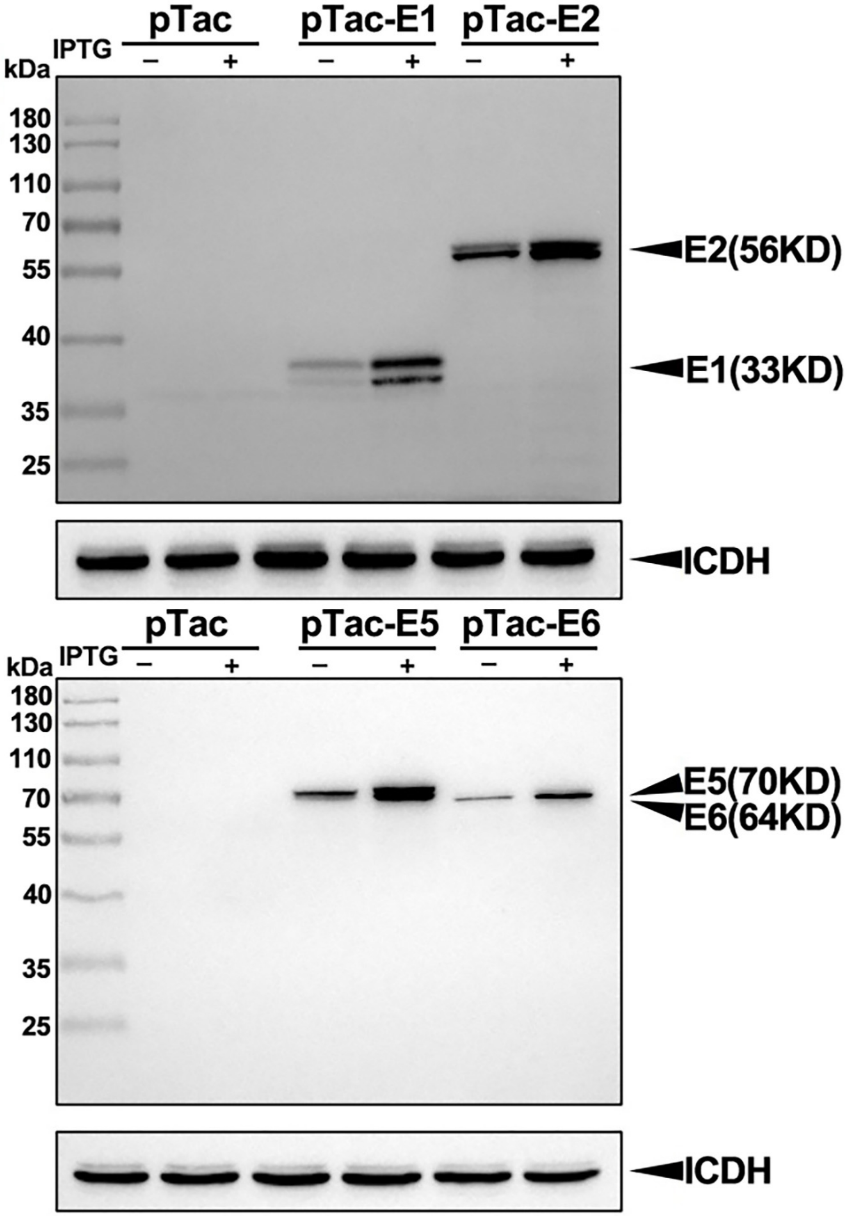

Supplement: Supplementary file 8 [file Data_Sheet_2.PDF]
